# Disparities in minimally invasive surgery for elective inguinal hernia repair across Europe: secondary analysis of an international cohort study

**DOI:** 10.1093/bjsopen/zraf122

**Published:** 2025-11-04

**Authors:** Maria Picciochi, Alberto G Barranquero

**Affiliations:** Institute of Applied Health Research, University of Birmingham, Heritage Building, Mindelsohn Way, Birmingham B15 2TH, UK; Abdominal Wall Surgery Unit, General and Digestive Surgery Department, Hospital Universitari Arnau de Vilanova, Av. Alcalde Rovira Roure, 80, 25198 Lleida, Catalonia, Spain

## Abstract

**Background:**

Healthcare systems in Europe vary in funding, accessibility, and spending per capita, potentially influencing patient access to advanced surgical techniques. This study aimed to provide a snapshot of the utilization of minimally invasive surgery for elective inguinal hernia repair across Europe.

**Methods:**

This was a secondary analysis of an international, prospective observational study of inguinal hernia repairs conducted between 30 January and 21 May 2023. Adults undergoing elective inguinal hernia repair in Europe were included in the present analysis. The four European regions according to the United Nations geoscheme (Southern, Eastern, Northern, and Western Europe) were compared. A multilevel multivariable logistic regression model was used to explore factors associated with use of minimally invasive surgery.

**Results:**

A total of 8355 patients from 254 hospitals across 23 European countries were included: 5590 from Southern, 587 from Eastern, 1541 from Northern, and 637 from Western Europe. Most hospitals were public (88.8%) and tertiary level (49.9%). Patient and hernia characteristics were generally similar, except Western Europe reported higher rates of bilateral hernias (25.9% *versus* 14.1% overall). Minimally invasive surgery was performed in 26.0% of patients, 70.6% in Western, 37.9% in Northern, 46.5% in Eastern, and 15.4% in Southern Europe. Multivariable regression showed significant regional disparities. Multivariable regression also identified bilateral hernias (adjusted odds ratio 14.33 (95% confidence interval 11.76 to 17.47), surgeons with experience of ≥ 201 procedures (odds ratio 3.54, 2.75 to 4.54), and private hospitals (odds ratio 2.80, 1.03 to 7.65) as factors associated with greater use of minimally invasive surgery.

**Conclusion:**

Significant disparities in minimally invasive surgery for elective inguinal hernia repair exist across Europe. Targeted initiatives should especially prioritize Southern Europe to ensure equitable access to advanced techniques.

## Teaser

Minimally invasive surgery (MIS) utilization in elective inguinal hernia repair varies significantly across Europe. In this analysis of 8355 patients from 23 countries, the highest rate of adoption of MIS was in Western Europe (70.6%) and the lowest in Southern Europe (15.4%). Factors associated with increased MIS use included bilateral hernias, high-volume surgeons, and private hospitals, highlighting the need for targeted efforts to improve access, particularly in Southern Europe.

## Introduction

The rising global burden of hernia-related health challenges, combined with advancements in minimally invasive surgical techniques, underscores the need for a comprehensive understanding of access disparities, environmental impacts, and procedural variability across regions. Addressing these issues, the HIPPO (Hernias, Pathway, and Planetary Outcomes for Inguinal Hernia Surgery) study^1^, launched in early 2023, was a global, prospective cohort study involving 18 058 patients from 640 centres across 83 countries, making it one of the largest studies of its kind. Its findings aim to shape policy recommendations, improve surgical training, and promote sustainable operating practices worldwide, representing a crucial step toward equitable and environmentally conscious surgical care that aligns with the diverse needs and capacities of healthcare systems^1,2^.

The results of the HIPPO study have demonstrated that elective inguinal hernia repair is essential for avoiding emergency operations, which are associated with higher rates of bowel resection^1^. Additionally, low- and middle-income countries reported lower rates of mesh repair^3^, a technique currently recommended to reduce the risk of recurrence^4,5^. Furthermore, these countries exhibited lower rates of minimally invasive inguinal hernia operations, emphasizing the need to expand access to these technologies^3^. Current guidelines^5^ recommend a laparoendoscopic technique because of its association with lower postoperative pain and a reduced incidence of chronic pain in patients of both sexes with primary unilateral inguinal hernia, provided that a surgeon with specific expertise and sufficient local/national resources is available. Laparoendoscopic repair is also suggested for bilateral hernia, for active young patients, and for those with high preoperative pain levels^4^.

This secondary analysis within the HIPPO study, focused on Europe, not only provides a snapshot of the current state of hernia surgery but also offers critical insights for developing strategies to promote equitable, high-quality surgical care across the continent. Europe's healthcare systems exhibit remarkable diversity, shaped by varying levels of public funding, accessibility, and health spending per capita^6^. Northern European countries benefit from robust healthcare policies that prioritize widespread access to care and advanced techniques. In contrast, Eastern European countries report lower health expenditures per capita, reflecting limited public funding and greater reliance on out-of-pocket payments^6^. Using inguinal hernia as a tracer condition for access to high-quality surgery^1^, significant variability in laparoscopic hernia surgery rates is evident across Europe, ranging from 4.6% in Italy during the years 2015–2020^7^ to 60.8% in Denmark during 2011–2020^8^. However, most studies have focused on long timeframes or single countries.

The primary aim of this study was to evaluate the rates of minimally invasive elective inguinal hernia surgery across the European regions as defined by the United Nations geoscheme: Southern, Eastern, Northern, and Western Europe^9^. A secondary aim was to identify the factors influencing its use across these regions, examine the adoption of day-case surgery, and evaluate postoperative complications.

## Methods

This was a subanalysis of the HIPPO study, an international prospective, cohort study that included patients undergoing primary inguinal hernia repair. The study protocol is publicly available, was registered in ClinicalTrials.gov (NCT05748886) and has been reported fully^1^. The HIPPO study comprised a global analysis of inguinal hernia treatment pathways, encompassing both elective and emergency procedures, as well as paediatric and adult primary repairs, aimed at evaluating reliance on emergency systems, the use of mesh-based repair, and access to minimally invasive surgery (MIS) across countries with varying income levels^1,3^. Any hospital performing inguinal hernia repair was eligible to take part. Data were collected prospectively between 30 January and 21 May 2023, from all consecutive patients undergoing inguinal hernia repair. This analysis is reported in line with STROBE guidelines^10^.

### Study population and setting

This analysis included adults, aged ≥ 16 years, undergoing elective primary inguinal hernia repair in hospitals in Europe. Recurrent inguinal hernias, operations through midline incisions, patients in whom no hernia was identified during surgery, and patients for whom the inguinal hernia repair was not the primary procedure were excluded. Patients were compared across the four European regions as defined by the United Nations geoscheme: Southern, Northern, Eastern, and Western Europe^9^.

### Data collection

Data were collected prospectively using a standardized protocol and recorded securely on the REDCap platform hosted at the University of Birmingham. Variables captured included patient demographics (age, sex, co-morbidities, American Society of Anesthesiologists (ASA) fitness grade), hernia characteristics, and procedural details such as surgical approach (open, laparoscopic, or robotic), and type of anaesthesia. Postoperative complications were evaluated at 30 days and categorized using the Clavien–Dindo classification system^11^. Each participating centre contributed data with completeness of at least 95%.

### Ethics and governance

Ethical approvals for the study were obtained from each participating centre according to local and national regulations. No changes to standard clinical care were implemented during the study; therefore, the study was registered as clinical audit or service evaluation in some centres where this was allowed (for example UK).

### Outcomes

The primary outcome of this analysis was use of MIS, defined as laparoscopic or robotic procedures, and included converted operations. Secondary outcomes included 30-day postoperative complications, graded according to the Clavien–Dindo classification^11^, surgical site infection (SSI), using the Centers for Disease Control definition^12^, and adoption of day-case surgery.

### Statistical analysis

Descriptive statistics were used to summarize baseline characteristics, with continuous variables reported as mean(standard deviation, s.d.), and categorical variables as frequencies and percentages. Differences between regions were tested using χ^2^ tests for categorical variables and Student's *t* test for continuous variables.

A multivariable multilevel logistic regression model was conducted to evaluate the influence of European regions on MIS use, adjusting for other factors that were preplanned based on previous literature^3^, and included: hospital funding, hospital type, patient sex, patient's indication for surgery, hernia size, and hernia site. To account for clustering effects, the hospital was included as a random effect in the model. The other outcomes are described as frequency and proportions.

To explore variation in complications across different regions, a multilevel logistic regression model was used. This model included hospitals as a random effect, variables previously shown to be associated with 30-day postoperative complications^1^, and other clinically plausible factors such as surgical experience and hospital funding.

All statistical analyses were undertaken using R statistical software version 4.0.3 (R Foundation for Statistical Computing, Vienna, Austria). All tests were two-tailed, with statistical significance set at *P* < 0.050.

## Results

**Fig. 1:**
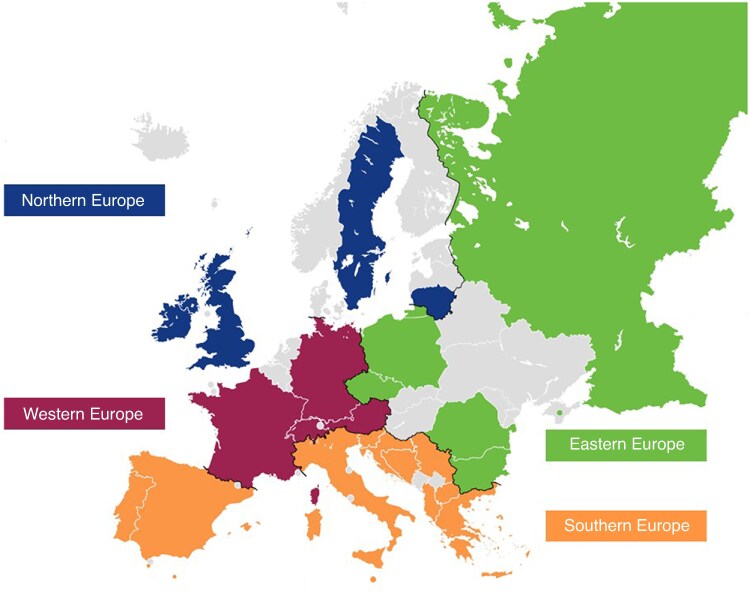
European regions based on the United Nations geoscheme Countries participating in the study are highlighted in colour.

The original HIPPO study collected data from 18 058 patients across 640 hospitals in 83 countries. In this subanalysis, 8355 patients underwent elective inguinal hernia repair in 254 hospitals from 23 European countries. The majority of patients were from Southern Europe (5590 patients), followed by Northern Europe (1541 patients), Western Europe (637 patients), and Eastern Europe (587 patients). Participant countries from different regions are displayed in *Fig. 1*. The distribution of participating hospitals and enrolled patients by country and European region is shown in *Table S1*.

### Baseline characteristics

Baseline characteristics are summarized in *Table 1*. Overall, the mean(s.d.) age of the included patients was 61.8(14.6) years. Men constituted the vast majority across all European regions, representing 91.0% of the total sample (7603 patients). The prevalence of significant co-morbidities (grade ASA III–IV) showed minor regional differences, with slightly higher rates in Western Europe (136 of 637, 21.4%) and Eastern Europe (304 of 1541, 19.7%).

**Table 1:** Baseline characteristics

	Southern Europe (*n* = 5590)	Eastern Europe (*n* = 587)	Northern Europe (*n* = 1541)	Western Europe (*n* = 637)	Total (*n* = 8355)
Age (years), mean(s.d.)	62.4(14.1)	58.9(15.0)	61.3(15.9)	60.5(15.4)	61.8(14.6)
**Sex**					
Male	5044 (90.2%)	540 (92.2%)	1434 (93.1%)	585 (91.8%)	7603 (91.0%)
Female	546 (9.8%)	46 (7.8%)	107 (6.9%)	52 (8.2%)	751 (9.0%)
**ASA fitness grade**					
I–II	4631 (82.8%)	486 (82.8%)	1217 (79.0%)	498 (78.2%)	6832 (81.8%)
III–V	930 (16.6%)	98 (16.7%)	304 (19.7%)	136 (21.4%)	1468 (17.6%)
Not recorded	29 (0.5%)	3 (0.5%)	20 (1.3%)	3 (0.5%)	55 (0.7%)
Missing	0 (0%)	0 (0%)	0 (0%)	0 (0%)	0 (0%)
**Co-morbidities**					
0	4146 (74.2%)	441 (75.3%)	1114 (72.3%)	477 (74.9%)	6178 (74.0%)
1	1079 (19.3%)	105 (17.9%)	311 (20.2%)	113 (17.7%)	1608 (19.2%)
2	285 (5.1%)	27 (4.6%)	89 (5.8%)	32 (5.0%)	433 (5.2%)
≥ 3	78 (1.4%)	13 (2.2%)	26 (1.7%)	15 (2.4%)	132 (1.6%)
Missing	2 (0.0%)	0 (0%)	1 (0.1%)	0 (0%)	3 (0.0%)
**Hernia size**					
Limited to inguinal region	4706 (84.2%)	505 (86.0%)	1297 (84.2%)	581 (91.2%)	7089 (84.8%)
Limited to scrotum	821 (14.7%)	76 (12.9%)	238 (15.4%)	54 (8.5%)	1189 (14.2%)
Extend to mid-thigh or beyond	63 (1.1%)	6 (1.0%)	6 (0.4%)	2 (0.3%)	77 (0.9%)
Missing	0 (0%)	0 (0%)	0 (0%)	0 (0%)	0 (0%)
**Indication**					
Asymptomatic	804 (14.4%)	71 (12.1%)	131 (8.5%)	71 (11.1%)	1077 (12.9%)
Symptomatic	4786 (85.6%)	516 (87.9%)	1410 (91.5%)	566 (88.9%)	7278 (87.1%)
Missing	0 (0%)	0 (0%)	0 (0%)	0 (0%)	0 (0%)
**Hernia site**					
Unilateral	4814 (86.1%)	526 (89.6%)	1366 (88.6%)	472 (74.1%)	7178 (85.9%)
Bilateral	776 (13.9%)	61 (10.4%)	175 (11.4%)	165 (25.9%)	1177 (14.1%)
Missing	0 (0%)	0 (0%)	0 (0%)	0 (0%)	0 (0%)

Values are *n* (%) unless otherwise stated. s.d., Standard deviation; ASA, American Society of Anesthesiologists.

Most patients (7278 of 8355, 87.1%) were symptomatic at diagnosis, with 91.5% of patients from Northern Europe (1410 of 1541) reporting symptoms. Asymptomatic patients were more common in Southern Europe (804 of 5590, 14.4%). Unilateral inguinal hernias were the most common presentation across all regions, accounting for 85.9% of patients (7178 of 8355), whereas bilateral hernias represented 14.1% (1177 of 8355). Patients from Western Europe had the highest prevalence of bilateral hernias (165 of 637, 25.9%).

### Hospital characteristics

Hospital characteristics are outlined in *Table 2*. Hospitals participating in the study varied substantially in type and infrastructure, and funding varied significantly across regions. Public hospitals comprised the majority, particularly in Northern Europe (1519 of 1541, 98.6%). On the contrary, private hospitals were most prevalent in Eastern Europe (254 of 587, 43.3%), indicating greater integration of private sector funding in healthcare delivery. Mixed-funding models were observed primarily in Western Europe (14.4%).

**Table 2:** Hospital characteristics

	Southern Europe (*n* = 5590)	Eastern Europe (*n* = 587)	Northern Europe (*n* = 1541)	Western Europe (*n* = 637)	Total (*n* = 8355)
**Hospital funding**					
Public	5075 (90.8%)	333 (56.7%)	1519 (98.6%)	496 (77.9%)	7423 (88.8%)
Public–private	377 (6.7%)	0 (0%)	0 (0%)	92 (14.4%)	469 (5.6%)
Private	115 (2.1%)	254 (43.3%)	0 (0%)	49 (7.7%)	418 (5.0%)
Missing	23 (0.4%)	0 (0%)	22 (1.4%)	0 (0%)	45 (0.5%)
**Hospital type**					
Primary	592 (10.6%)	61 (10.4%)	164 (10.6%)	111 (17.4%)	928 (11.1%)
Secondary	2282 (40.8%)	274 (46.7%)	537 (34.8%)	117 (18.4%)	3210 (38.4%)
Tertiary	2693 (48.2%)	252 (42.9%)	818 (53.1%)	409 (64.2%)	4172 (49.9%)
Missing	23 (0.4%)	0 (0%)	22 (1.4%)	0 (0%)	45 (0.5%)
**Emergency surgery available**					
No	38 (0.7%)	45 (7.7%)	31 (2.0%)	0 (0%)	114 (1.4%)
Yes—assessment only and transfer for surgery	179 (3.2%)	0 (0%)	0 (0%)	0 (0%)	179 (2.2%)
Yes—assessment and emergency surgery during daytime	163 (2.9%)	49 (8.3%)	133 (8.8%)	19 (3.0%)	364 (4.4%)
Yes—assessment and emergency surgery available 24 h	5187 (93.2%)	493 (84.0%)	1355 (89.2%)	618 (97.0%)	7653 (92.1%)
**Day-case surgery unit available**					
Yes	5195 (93.3%)	347 (59.1%)	1519 (100%)	630 (98.9%)	7691 (92.6%)
No	372 (6.7%)	240 (40.9%)	0 (0%)	7 (1.1%)	619 (7.4%)

Values are *n* (%).

The availability of day-case surgery units varied significantly across regions. Northern and Western Europe had the highest rates of treatment in these facilities, at 100% (all 1519 patients) and 98.9% (630 of 637), respectively. In contrast, only 59.1% of patients in Eastern Europe (347 of 587) had access to day-case surgery facilities.

### Primary outcomes

*Table 3* shows a summary of surgical outcomes. The overall rate of MIS in elective inguinal hernia surgery was 26% (2169 of 8355). The highest rates were observed in Western Europe (450 of 637, 70.6%), followed by Eastern Europe (273 of 587, 46.5%) and Northern Europe (584 of 1541, 37.9%). The rate of MIS in Southern Europe was limited to 15.4% (862 of 5590). Robotic surgery was performed in 11% of patients in Western Europe (70 of 637), compared with a lower rate of 0.9% (14 of 1541) in Northern Europe and 0.5% (26 of 5590) in Southern Europe. None of the patients from Eastern Europe underwent robotic inguinal hernia surgery in this study.

**Table 3:** Surgical outcomes

	Southern Europe (*n* = 5590)	Eastern Europe (*n* = 587)	Northern Europe (*n* = 1541)	Western Europe (*n* = 637)	Total (*n* = 8355)
**Surgical approach**					
MIS/MIS converted	862 (15.4%)	273 (46.5%)	584 (37.9%)	450 (70.6%)	2169 (26.0%)
Open	4728 (84.6%)	314 (53.5%)	957 (62.1%)	187 (29.4%)	6186 (74.0%)
Missing	0 (0%)	0 (0%)	0 (0%)	0 (0%)	0 (0%)
**Approach (detailed)**					
Laparoendoscopic	813 (14.5%)	265 (45.1%)	553 (35.9%)	374 (58.7%)	2005 (24.0%)
Laparoendoscopic converted to open	23 (0.4%)	8 (1.4%)	17 (1.1%)	6 (0.9%)	54 (0.6%)
Robotic	26 (0.5%)	0 (0%)	14 (0.9%)	70 (11.0%)	110 (1.3%)
Open	4728 (84.6%)	314 (53.5%)	957 (62.1%)	187 (29.4%)	6186 (74.0%)
Missing	0 (0%)	0 (0%)	0 (0%)	0 (0%)	0 (0%)
**Mesh used**					
Yes	5533 (99.0%)	576 (98.1%)	1527 (99.1%)	636 (99.8%)	8272 (99.0%)
No	57 (1.0%)	11 (1.9%)	14 (0.9%)	1 (0.2%)	83 (1.0%)
Missing	0 (0%)	0 (0%)	0 (0%)	0 (0%)	0 (0%)
**Surgical technique (mesh-based)**					
Transabdominal preperitoneal repair	566 (10.2%)	237 (41.1%)	404 (26.5%)	263 (41.4%)	1470 (17.8%)
Totally extraperitoneal repair	260 (4.7%)	23 (4.0%)	137 (9.0%)	180 (28.3%)	600 (7.3%)
Lichtenstein	3752 (67.8%)	309 (53.6%)	886 (58.0%)	177 (27.8%)	5124 (61.9%)
Plug and patch	796 (14.4%)	2 (0.3%)	30 (2.0%)	5 (0.8%)	833 (10.1%)
PHS™ † (bilayer)	17 (0.3%)	1 (0.2%)	5 (0.3%)	9 (1.4%)	32 (0.4%)
Transrectal preperitoneal	13 (0.2%)	0 (0%)	2 (0.1%)	1 (0.2%)	16 (0.2%)
Transinguinal preperitoneal	7 (0.1%)	0 (0%)	36 (2.4%)	1 (0.2%)	44 (0.5%)
Other	122 (2.2%)	4 (0.7%)	27 (1.8%)	0 (0%%)	153 (1.8%)
Missing	0 (0%)	0 (0%)	0 (0%)	0 (0%)	0 (0%)
Mesh type*					
Permanent synthetic	5105 (92.3%)	497 (86.3%)	1323 (86.6%)	556 (87.4%)	7481 (90.4%)
Absorbable synthetic	328 (5.9%)	75 (13.0%)	120 (7.9%)	48 (7.5%)	571 (6.9%)
Biological	1 (0.0%)	3 (0.5%)	5 (0.3%)	0 (0%)	9 (0.1%)
Composite	98 (1.8%)	1 (0.2%)	79 (5.2%)	31 (4.9%)	209 (2.5%)
Missing	1 (0.0%)	0 (0%)	0 (0%)	1 (0.2%)	0 (0%)
Mesh fixation*					
Not fixed	726 (13.1%)	168 (29.2%)	216 (14.1%)	160 (25.2%)	1270 (15.4%)
Absorbable suture	1785 (32.3%)	85 (14.8%)	163 (10.7%)	40 (6.3%)	2073 (25.1%)
Non-absorbable suture	2524 (45.6%)	135 (23.4%)	850 (55.7%)	220 (34.6%)	3729 (45.1%)
Glue (fibrin/histoacryl)	219 (4.0%)	133 (23.1%)	65 (4.3%)	182 (28.6%)	599 (7.2%)
Tackers	278 (5.0%)	54 (9.4%)	233 (15.3%)	34 (5.3%)	599 (7.2%)
Missing	1 (0.0%)	1 (0.2%)	0 (0%)	0 (0%)	0 (0%)
**Contamination**					
Clean	5572 (99.7%)	569 (96.9%)	1531 (99.4%)	635 (99.7%)	8307 (99.4%)
Clean-contaminated	14 (0.3%)	18 (3.1%)	9 (0.6%)	2 (0.3%)	43 (0.5%)
Contaminated	3 (0.1%)	0 (0%)	1 (0.1%)	0 (0%)	4 (0.0%)
Dirty	1 (0.0%)	0 (0%)	0 (0%)	0 (0%)	1 (0.0%)
Missing	0 (0%)	0 (0%)	0 (0%)	0 (0%)	0 (0%)
**Bowel resection**					
No	5566 (99.6%)	583 (99.3%)	1535 (99.6%)	634 (99.5%)	8318 (99.6%)
Yes	24 (0.4%)	4 (0.7%)	6 (0.4%)	3 (0.5%)	37 (0.4%)
**Type of anaesthesia**					
General	2471 (44.2%)	367 (62.5%)	1292 (83.8%)	602 (94.5%)	4732 (56.6%)
Local	871 (15.6%)	23 (3.9%)	135 (8.8%)	20 (3.1%)	1049 (12.6%)
Regional block	92 (1.6%)	0 (0%)	17 (1.1%)	3 (0.5%)	112 (1.3%)
Spinal	2156 (38.6%)	197 (33.6%)	97 (6.3%)	12 (1.9%)	2462 (29.5%)
**Primary operator**					
Senior surgeon (consultant or attending)	4082 (73.0%)	399 (68.0%)	1209 (78.5%)	509 (79.9%)	6199 (74.2%)
Trainee surgeon	1499 (26.8%)	188 (32.0%)	329 (21.3%)	128 (20.1%)	2144 (25.7%)
Non-surgeon, medical practitioners	9 (0.2%)	0 (0%)	3 (0.2%)	0 (0%)	12 (0.1%)
Missing	0 (0%)	0 (0%)	0 (0%)	0 (0%)	0 (0%)
**Experience of primary operator (no. of procedures)**					
0–50	990 (17.7%)	86 (14.7%)	188 (12.2%)	90 (14.1%)	1354 (16.2%)
51–200	1475 (26.4%)	176 (30.0%)	429 (27.8%)	186 (29.2%)	2266 (27.1%)
≥ 201	3125 (55.9%)	325 (55.4%)	918 (59.6%)	360 (56.5%)	4728 (56.6%)
Missing	0 (0%)	0 (0%)	6 (0.4%)	1 (0.2%)	7 (0.1%)

Values are *n* (%). *Assessed only in patients in whom a mesh was used. MIS, minimally invasive surgery. † PHS™, Polypropylene Hernia System (Johnson & Johnson, New Brunswick, New Jersey, United States)

The use of MIS by specific patient characteristics is summarized in *Table S2*. Among women, the rate ranged from 17.0% (93 of 546) in Southern Europe to 80.8% (42 of 52) in Western Europe. For bilateral hernias, similar rates were observed across regions, with rates always above 50%.

The most common MIS procedure performed was transabdominal preperitoneal (TAPP) repair (overall 1470 of 8355, 17.8%) whereas the most common open procedure was the Lichtenstein technique (overall 5124 of 8355, 61.9%). Only 37 patients (0.4%) underwent bowel resection at the time of surgery; the rate also varied across regions.

Overall, 6199 of 8355 interventions (74.2%) were undertaken by senior surgeons. The proportion of operations performed by trainee surgeons in Eastern Europe (188 of 587, 32%) was higher than the overall rate of 25.7% (2144 of 8355).

**Fig. 2:**
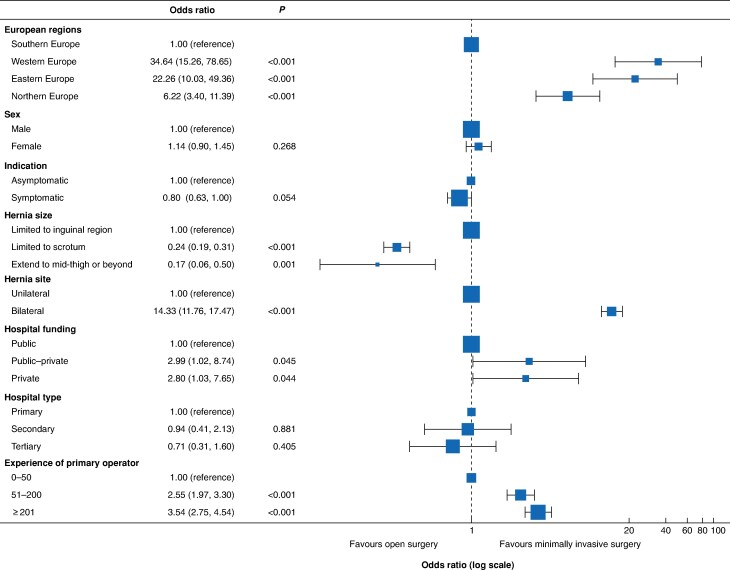
Multivariable regression model of risk factors for minimally invasive inguinal hernia repair Odds ratios are shown with 95% confidence intervals. The dashed line indicates an odds ratio value of 1.00.

European regions were associated with the use of MIS, and this variation was observed in the multivariable logistic regression model (*Fig. 2*). Patients who had surgery in Northern, Eastern, and Western Europe all had a greater likelihood of undergoing MIS. Having surgery in a private hospital (odds ratio (OR) 2.80, 95% confidence interval (c.i.) 1.03 to 7.65) or carried out by surgeons with experience of ≥ 201 procedures (OR 3.54, 2.75 to 4.54), or having bilateral hernias (OR 14.33, 11.76 to 17.47) were also associated with higher rates of MIS. Patients with hernias extending to the scrotum and beyond had a lower likelihood of undergoing MIS.

### Secondary outcomes

Secondary outcomes are summarized in *Table 4*. Elective inguinal hernia repair was performed as a day-case procedure in 58% of the sample evaluated (4847 of 8355), with higher rates in Northern Europe (1229 of 1541, 79.8%) and lower rates in Eastern Europe (181 of 587, 30.8%).

**Table 4:** Postoperative outcomes

	Southern Europe (*n* = 5590)	Eastern Europe (*n* = 587)	Northern Europe (*n* = 1541)	Western Europe (*n* = 637)	Total (*n* = 8355)
**Day-case surgery**					
No	2413 (43.2%)	406 (69.2%)	311 (20.2%)	359 (56.4%)	3489 (41.8%)
Yes	3160 (56.5%)	181 (30.8%)	1229 (79.8%)	277 (43.5%)	4847 (58.0%)
Missing	17 (0.3%)	0 (0%)	1 (0.1%)	1 (0.2%)	19 (0.2%)
**Clavien–Dindo grade**					
0 (no complications)	4903 (87.7%)	485 (82.6%)	1425 (92.5%)	561 (88.1%)	7374 (88.3%)
I–II	641 (11.5%)	94 (16.0%)	101 (6.6%)	62 (9.7%)	898 (10.7%)
III–V	33 (0.6%)	8 (1.4%)	14 (0.9%)	13 (2.0%)	68 (0.8%)
Missing	13 (0.2%)	0 (0%)	1 (0.1%)	1 (0.2%)	15 (0.2%)
**Surgical site infection**					
No	5437 (97.5%)	566 (96.4%)	1520 (98.7%)	627 (98.6%)	8150 (97.7%)
Yes	140 (2.5%)	21 (3.6%)	20 (1.3%)	9 (1.4%)	190 (2.3%)
**Reoperation**					
No	5560 (99.7%)	585 (99.7%)	1528 (99.2%)	629 (98.9%)	8302 (99.5%)
Yes	17 (0.3%)	2 (0.3%)	12 (0.8%)	7 (1.1%)	38 (0.5%)

Values are *n* (%).

The overall complication rate was 11.6% (966 of 8355), and interventions in Eastern Europe showed a higher rate of complications (102 of 587, 17.4%). A similar increase was observed in the proportion of grade I–II complications, according to the Clavien–Dindo classification (16% (94 of 587) *versus* 10.7% (898 of 8355)), and in the SSI rates (3.6% (21 of 587) *versus* 2.3% (190 of 8355)).

Nevertheless, the multivariable regression model of risk factors for postoperative complications (*Table S3*) showed that complication risk was not associated with European region, hospital funding, or experience level of the primary operator. Instead, higher risk was linked to an ASA grade of III–V (OR 1.40, 95% c.i. 1.17 to 1.67; *P* < 0.001), procedures classified as clean-contaminated, contaminated, or dirty (OR 2.87, 1.30 to 6.35; *P* = 0.009), and the need for bowel resection (OR 2.38, 1.00 to 5.63; *P* = 0.049). In contrast, day-case surgery was associated with a reduced risk of 30-day postoperative complications (OR 0.38, 0.32 to 0.46; *P* < 0.001).

## Discussion

The findings of this study support the initial hypothesis that the rate of elective MIS for inguinal hernia repair varies across Europe. This unplanned subgroup analysis of the original global HIPPO study^1^ provides valuable insights into current trends in inguinal hernia repair across Europe. Accurate, up-to-date data on the precise rates of MIS in inguinal hernia repair can be challenging to obtain, with true rates available only from some registry-based studies, although these were often historical and may not truly reflect current practice. In contrast, the present study provides a timely, high-quality snapshot of inguinal hernia repair practices across Europe between 30 January and 21 May 2023.

A significant strength of this study lies in its well conducted reanalysis of pre-existing HIPPO data. These were high-quality data collected using established research methodology that has been demonstrated to be reliable and robust. The analysis plan, although not completed *a priori*, was established before completion of the statistical testing. The HIPPO data were purely observational and, although they were collected at a local level, the impact of potential observer bias was deemed to be low.

One major limitation of the study is the variation in patient representation from different countries and healthcare settings across the different European regions, which may have influenced the final MIS rates. In the Northern Europe group, the absence of participating centres from Denmark, Norway, and Finland may have led to an underestimation of the use of MIS for inguinal hernia repair. The published literature demonstrates a high rate of use of MIS in these countries, with 60.8% of inguinal hernia repairs in Denmark during 2011–2020 being performed using the TAPP technique^8^. Sweden, the only Nordic country represented in the present study, reported that, in 2021, laparoscopic techniques for inguinal hernia repair were used in approximately 40% of men and up to 80% of women^13^. The Northern Europe group analysed here consisted predominantly of patients from the UK. This limits the ability to compare the present results with previously published literature, as most of the available data are outdated. For example, a previous study^14^ reported a 23.2% MIS rate in England during 2011–2017, which may not reflect current trends.

The data from Eastern Europe may have been disproportionately affected by the high proportion of patients treated in privately funded hospitals (43.3% *versus* 5.0% overall). Nationwide analysis from Romania^15^ showed that MIS repair was performed in 12.6% of patients in the public system between 2019 and 2021, compared with 50.6% of those in the private system. This is consistent with data reported elsewhere in the literature, showing increased odds of MIS for inguinal hernia repair in private hospitals^3,16,17^.

Having addressed the limitations of the study, it is relevant to highlight that the present results demonstrate a higher rate of MIS in Western Europe (70.6%) than documented previously. Previous studies reported a MIS rate of 56.5% in France during 2015–2021^18^, and 58.6% for unilateral inguinal hernia in the Herniamed Registry (Germany, Austria, and Switzerland) during 2010–2020^19^.

In contrast, the Southern Europe group had lower rates of MIS for inguinal hernia repair (15.4%). This group had a large representation from Italy and Spain, where laparoscopic inguinal hernia repair accounted for only 4.5% of procedures in Italy (2015–2020)^7^ and 5.7% in Spain (2016–2018)^16^. Data from other countries in this region are scarce, but multicentre data identified a MIS rate of 36.4% in the Republic of Northern Macedonia^20^ and 10.3% in Slovenia^21^. This region includes Southeastern European countries, which have fewer economic resources than other high-income European nations^22^, leading to limited access to MIS equipment and issues with mesh availability. Therefore, despite the variation in patient numbers, the present findings are likely to reflect an accurate rate of MIS for inguinal hernia repair in this region.

There has been a steady shift over time from open hernia repair to MIS techniques^7,13,15^. The use of MIS is supported by the latest clinical guidelines, with Europena Hernia Society guidelines recommending laparoscopic techniques for the primary repair of unilateral inguinal hernias^5^, although the decision between totally extraperitoneal repair or TAPP repair should be based on the surgeon's skills, education, and experience^4^. However, these guidelines state that specific patient and hernia characteristics still warrant use of Lichtenstein or open preperitoneal mesh techniques^5^. Patients with multiple co-morbidities or recurrent hernias^23^ or inguinoscrotal hernias^24^ are more likely to undergo open hernia repair. The limited adoption of MIS may reflect surgeon preference^16^ despite adequate surgical expertise, insufficient training among those with limited procedural experience, and concerns about direct costs.

Inguinal hernia guidelines also emphasize the importance of local resources when considering a minimally invasive repair^5^. In Eastern Europe, access to advanced surgical techniques and medical devices for inguinal hernia repair likely depends on out-of-pocket patient payments in the private sector^6^. In some Southern European countries, where public health expenditure is relatively limited compared with that in the rest of the European Union^6^, policymakers often prioritize a higher volume of procedures at a lower direct cost^16^. Greater emphasis on early recovery and improved quality of life could help justify increased resource allocation for advanced technologies in inguinal hernia repair.

The evaluation of 30-day postoperative complications indicates that elective inguinal hernia surgery in Europe is associated with a low risk of severe morbidity, with no significant differences observed across European regions. The risk factors for postoperative complications identified in this study closely align with those known to predict mortality following urgent inguinal hernia surgery^25^. In this context, with the exception of selected high-risk patients, day-case surgery should be more common than reflected in the present data. Although the proportion of patients undergoing outpatient procedures has increased over time^26^, some healthcare systems still rely heavily on inpatient care^6^, and day-case surgery units remain underdeveloped, particularly in Eastern Europe. Besides, the differences in hospital stay between open and minimally invasive inguinal hernia repair are less pronounced than in other surgical fields, such as colonic surgery^27^ or cholecystectomies^28^, where laparoscopy has long been the standard. Expanding the use of day-case surgery could help reduce healthcare costs while maintaining high-quality outcomes^29^, potentially offsetting the increased expenses associated with minimally invasive techniques^30^.

This study also highlighted areas that warrant further observation. Notably, the rate of use of the plug-and-patch technique in Southern Europe was relatively high, accounting for 14.4% of all repairs in the region (796 of 5590), despite recommendations against it owing to the excessive use of foreign material and the associated risk of plug erosion^4^. Additionally, 12.9% of all repairs in the study (1077 of 8355) were performed on asymptomatic hernias. Surgical intervention in such instances should be carefully discussed with the patient, as watchful waiting for asymptomatic inguinal hernias can be suggested in men^4,5^. Further research is needed in this area to prevent unnecessary operations that may strain healthcare systems^31^.

The increasing rates of MIS for inguinal hernia repair and recent clinical guidelines make it imperative that thorough consideration is given to surgical education, and enhancing the experience of residents and fellows in minimally invasive procedures. A minimum of 60 procedures during surgical residency is considered necessary to safely perform open anterior mesh repair for groin hernia without supervision^32^. Limited training opportunities in MIS repair are often a limiting factor, despite the requirement for graduating residents to be competent in performing these techniques^33^. The disparity between different European regions has been recognized by some surgical societies, leading to the creation of the Forward Program of the European Association of Endoscopic Surgeons, which aims to support the technologically less advanced Southeastern Europe by providing education in MIS surgery for young surgeons^34^.

In this setting, robot-assisted surgery may offer a shorter learning curve for inguinal hernia repair with more active participation than laparoscopic procedures^35^, while also providing an opportunity to convert open operations into minimally invasive procedures, potentially benefitting patient outcomes^36^. The rate of robot-assisted procedures remains low in Europe, reaching only 11% in Western Europe and less than 1% in other regions in the present study. Despite its benefits, the higher cost of robot-assisted surgery compared with laparoscopic techniques (€2612 *versus* €1963)^37^, along with longer operating times^38^, may limit its accessibility in certain healthcare systems^39^, potentially creating new inequities between European regions.

Significant disparities in MIS adoption for elective inguinal hernia repair exist across Europe. Targeted initiatives should especially prioritize Southern Europe to ensure more equitable access to advanced surgical techniques and ensure that practices align with the latest European clinical guidelines.

## Supplementary Material

zraf122_Supplementary_Data

## Data Availability

Anonymized data are available upon request from the writing group and successful completion of a data-sharing agreement through an Application Programming Interface linked to the REDCap data server hosted at the University of Birmingham, Birmingham, UK.
