# Supplementary material for: Disparities in minimally invasive surgery for elective inguinal hernia repair across Europe: secondary analysis of an international cohort study
Source: BJS Open. 2025 Nov 4;9(6):zraf122. doi: 10.1093/bjsopen/zraf122 (PMC12586846; doi:10.1093/bjsopen/zraf122)
Supplement: zraf122_Supplementary_Data [file zraf122_supplementary_data.docx]

**Disparities in Minimally Invasive Surgery for Elective Inguinal Hernia Repair Across Europe: Secondary Analysis of an International Cohort Study**

National Institute for Health and Care Research (NIHR) Global Research Health Unit on Global Surgery

**Corresponding authors:**

Dr Maria Picciochi, NIHR Global Health Research Unit on Global Surgery, Institute of Applied Health Sciences, University of Birmingham, Birmingham B15 2TH, UK

[m.picciochi@bham.ac.uk](mailto:m.picciochi@bham.ac.uk) ORCID: 0000-0003-0397-5076

and

Dr Alberto G Barranquero, Abdominal Wall Surgery Unit, General and Digestive Surgery Department, Hospital Universitari Arnau de Vilanova, Lleida, Catalonia, Spain

[agbarranquero.es@gmail.com](mailto:agbarranquero.es@gmail.com) ORCID: 0000-0001-8510-8210

**Supplementary Materials – Index**

| **Supplementary Results** |  |
| --- | --- |
| Table S1. Distribution of participating hospitals and enrolled patients by country and European region | *pag. 2* |
| Table S2. Use of minimally invasive surgery by patient and hernia characteristics | *pag. 3* |
| Table S3. Univariable analysis and multivariable regression model of risk factors for  30-day postoperative complications | *pag. 4* |
| **Supplementary Appendixes** |  |
| List of authors | *pag. 5-21* |

**Supplementary Results**

Table S1. Distribution of participating hospitals and enrolled patients by country and European region

| **European region** | **Participant countries** | **Hospitals** | **Patients** |
| --- | --- | --- | --- |
| Southern Europe | Albania | 2 | 10 |
|  | Bosnia and Herzegovina | 6 | 236 |
|  | Croatia | 2 | 76 |
|  | Greece | 30 | 648 |
|  | Italy | 64 | 1950 |
|  | Malta | 1 | 71 |
|  | Portugal | 21 | 725 |
|  | Republic of North Macedonia | 1 | 0 |
|  | Serbia | 1 | 33 |
|  | Slovenia | 1 | 8 |
|  | Spain | 24 | 1833 |
| Eastern Europe | Bulgaria | 6 | 40 |
|  | Czech Republic | 6 | 118 |
|  | Poland | 8 | 308 |
|  | Romania | 5 | 25 |
|  | Russian Federation* | 5 | 86 |
| Northern Europe | Ireland | 2 | 5 |
|  | Lithuania | 3 | 192 |
|  | Sweden | 2 | 54 |
|  | United Kingdom | 47 | 1290 |
| Western Europe | Austria | 5 | 95 |
|  | France | 3 | 112 |
|  | Germany | 11 | 160 |
|  | Switzerland | 6 | 270 |

* One hospital attributed to the Russian Federation is located in the territory of Crimea, which is disputed between Ukraine and the Russian Federation; it is represented in Figure 1 by a green dot.

Table S2. Use of minimally invasive surgery by patient and hernia characteristics

|  | **Southern Europe** | **Eastern Europe** | **Northern Europe** | **Western Europe** | **Total** |
| --- | --- | --- | --- | --- | --- |
|  | n (%) | n (%) | n (%) | n (%) | n (%) |
| **Female patients** | 546 | 46 | 107 | 52 | 751 |
| MIS/MIS converted | 93 (17.0%) | 17 (37.0%) | 40 (37.4%) | 42 (80.8%) | 192 (25.6%) |
| Open | 453 (83.0%) | 29 (63.0%) | 67 (62.6%) | 10 (19.2%) | 559 (74.4%) |
| **Bilateral hernia** | 776 | 61 | 175 | 165 | 1177 |
| MIS/MIS converted | 420 (54.1%) | 55 (90.2%) | 136 (77.7%) | 143 (86.7%) | 754 (64.1%) |
| Open | 356 (45.9%) | 6 (9.8%) | 39 (22.3%) | 22 (13.3%) | 423 (35.9%) |

Data are presented as n (%).

MIS: minimally invasive surgery, which included laparoscopic and robotic approaches.

Table S3. Univariable analysis and multivariable regression model of risk factors for 30-day postoperative complications

|  | **No complications**  **n: 7374** | **Complications**  **n: 966** | **Univariable analysis** | **Multivariable regression model** |
| --- | --- | --- | --- | --- |
|  | **n (%)** | **n (%)** | **OR (95% CI, p-value)** | **OR (95% CI, p-value)** |
| **Europe regions** |  |  |  |  |
| Southern Europe | 4903 (87.9) | 674 (12.1) | - | - |
| Western Europe | 561 (88.2) | 75 (11.8) | 0.97 (0.75-1.25, p=0.830) | 0.85 (0.50-1.46, p=0.553) |
| Eastern Europe | 485 (82.6) | 102 (17.4) | 1.53 (1.21-1.91, p<0.001) | 1.03 (0.59-1.78, p=0.920) |
| Northern Europe | 1425 (92.5) | 115 (7.5) | 0.59 (0.48-0.72, p<0.001) | 0.89 (0.59-1.35, p=0.594) |
| **ASA groups** |  |  |  |  |
| ASA I-II | 6081 (89.2) | 738 (10.8) | - | - |
| ASA III-V | 1244 (84.8) | 223 (15.2) | 1.48 (1.25-1.73, p<0.001) | 1.40 (1.17-1.67, p<0.001) |
| Not recorded | 49 (90.7) | 5 (9.3) | 0.84 (0.29-1.92, p=0.713) | 1.19 (0.43-3.36, p=0.735) |
| **Hospital funding** |  |  |  |  |
| Public | 6559 (88.5) | 850 (11.5) | - | - |
| Public-Private | 426 (90.8) | 43 (9.2) | 0.78 (0.56-1.06, p=0.128) | 1.10 (0.54-2.22, p=0.802) |
| Private | 349 (83.5) | 69 (16.5) | 1.53 (1.16-1.98, p=0.002) | 1.09 (0.55-2.14, p=0.807) |
| **Contamination** |  |  |  |  |
| Clean | 7338 (88.5) | 954 (11.5) | - | - |
| Non-clean* | 36 (75.0) | 12 (25.0) | 2.56 (1.27-4.80, p=0.005) | 2.87 (1.30-6.35, p=0.009) |
| **Bowel resection** |  |  |  |  |
| No | 7346 (88.5) | 957 (11.5) | - | - |
| Yes | 28 (75.7) | 9 (24.3) | 2.47 (1.10-5.04, p=0.019) | 2.38 (1.00-5.63, p=0.049) |
| **Day-case** |  |  |  |  |
| No | 2901 (83.1) | 588 (16.9) | - | - |
| Yes | 4469 (92.2) | 378 (7.8) | 0.42 (0.36-0.48, p<0.001) | 0.38 (0.32-0.46, p<0.001) |
| **Experience of primary operator** |  |  |  |  |
| 0-50 procedures | 1182 (87.6) | 167 (12.4) | - | - |
| 51-200 procedures | 1960 (86.6) | 302 (13.4) | 1.09 (0.89-1.34, p=0.401) | 1.25 (0.99-1.58, p=0.060) |
| ≥201 procedures | 4225 (89.5) | 497 (10.5) | 0.83 (0.69-1.01, p=0.055) | 1.08 (0.86-1.35, p=0.496) |

*Non-clean includes clean-contaminated, contaminated and dirty surgeries.

**Supplementary Appendix: List of authors**

**Writing group:** AG Barranquero*, M Picciochi*, A Butyrskii, S Delibegovic, M Elhadi, G Gallo, E Gialamas, JG Goncalves-Nobre, N Gouvas, I Katsaros, S Lawday, H Lederhuber, MW Löffler, A Minaya Bravo, M Nikberg, I Negoi, SZY Ooi, F Pata, T Risteski, M Sampaio-Alves, G Tsoulfas, A Yiallourou, R Villalobos Mori.

*denotes joint first authors

**Study management group:** M Picciochi, AO Ademuyiwa, A Adisa, AE Agbeko, JA Calvache, D Chaudhry, R Crawford, AC Dawson, M Elhadi, A Ghaffar, D Ghosh, J Glasbey, PD Haque, E Harrison, A Isik, I Jakaityte, SK Kamarajah, O Kouli, I Lawani, S Lawani, V Ledda, E Li, J Martin, A Minaya Bravo, D Morton, D Nepogodiev, F Ntirenganya, O Omar, SZY Ooi, R Oppong, F Pata, A Ramos-De la Medina, M Sampaio-Alves, JFF Simoes, M Steinruecke, S Tabiri, A Bhangu.

**Data handling and management:** M Picciochi, R Acharya, D Badran, A Chaudhry, JG Goncalves-Nobre, RR Gujjuri, B Kadir, SR Knight, S Lawday, O Omar, KSY Ooi, R Ooi, C Varghese.

**Dissemination Committee** (listed by country)**:**

Albania: I Dajti; Algeria: Z Djama; Argentina: M Lucchini, RM Palacios Huatuco; Australia: K Atherton, AC Dawson, E Lun; Austria: F Aigner; Belgium: F Berrevoet; Benin: I Lawani, S Lawani, C Bokossa; Bosnia and Herzegovina: S Delibegovic; Bulgaria: M Slavchev; Burkina Faso: AF Sanon, A Sanou; Burundi: JB Gusa, JC Mbonicura; Cameroon: A Bang, O Gabom, C Nwegbu; Canada: A Brar, J Martin; Chile: MM Modolo, M Olivos; Colombia: JA Calvache; Croatia: J Mihanovic; Cyprus: N Gouvas, A Yiallourou; Czech Republic: B East; Dominican Republic: S Batista, R Rivas; Ecuador: EP Lincango; Egypt: S Emile; Ethiopia: AB Aregawi; France: AP Arnaud; Gabon: N Boumas; Georgia: Z Demetrashvili; Germany: H Lederhuber, MW Löffler; Ghana: AE Agbeko, NB Sam, S Tabiri, F Agyei, FE Gyamfi, S Mohammed; Greece: I Katsaros, G Tsoulfas; India: L Bains, J Dhiman, D Ghosh, PD Haque, A Suroy; Ireland: S Ramjit; Israel: G Marom; Italy: F Pata, G Gallo; Jordan: F Ayasra; Kazakhstan: I R Fakhradiyev; Kenya: IHS Hamdun; Kyrguzstan: A Iqbal; Liberia: E Mbanzabugabo; Libya: M Elhadi; Lithuania: A Gulla; Madagascar: L Samison; Malawi: M Nyirenda, R Nyirenda; Malaysia: AC Roslani; Mali: B Bengaly; Malta: J Psaila; Mexico: L Martinez, A Ramos-De la Medina; Namibia: PR Nashidengo; New Zealand: M McGuinnes, D Wright; Niger: A Ousseini; Nigeria: A Adisa, AO Ademuyiwa; Republic of North Macedonia: T Risteski; Oman: Z Al Balushi, B Dawud, A AlSharqi, F Ali; Pakistan: AU Qureshi; Palestine: H Abu-Arish; Paraguay: H Gomez-Fernandez; Philippines: JM Faylona, MD Sacdalan; Poland: W Krawczyk; Portugal: JG Goncalves-Nobre, M Sampaio-Alves, I Santos; Romania: I Negoi; Russian Federation: A Butyrskii; Rwanda: JC Allen, F Ntirenganya; Sierra Leone: I Fortune; Slovenia: J Kosir; South Africa: N Parker, K Chu; Spain: A Minaya Bravo; Sri Lanka: D Wickramasinghe, U Jayarajah; Sudan: M Elmujtaba; Sweden: M Nikberg; Switzerland: E Gialamas; Syria: M Alshaar; Tanzania: M Nkoronko; Turkey: A Isik; Uganda: I Mubesi; United States: J Ng-Kamstra; Venezuela: O Bahsas-Zaky.

**Hospital leads** (listed by country and city)**:**

Albania: E Agastra (Korca; Regional Hospital of Korca); I Dajti (Tirana; University hospital Koco Gliozheni).

Austria: I Königsrainer (Feldkirch; Landeskrankenhaus Feldkirch); F Aigner (Graz; Barmherzige Brüder Krankenhaus, Graz); S Mikalauskas (Graz; Medical University of Graz); L Havranek (Linz; Ordensklinikum Linz Elisabethinen); A Binder (Tulln; Universitätsklinikum Tulln).

Bosnia and Herzegovina: Z Matkovic (Doboj; Genera Hospital ‘Sveti aposto Luka’ Doboj); N Lalovic (Foča; University Hospital Foča); J Miskovic (Mostar; SKB University Clinical Hospital Mostar); M Salibašić (Sarajevo; Clinical Center University of Sarajevo); A Cerovac (Tešanj; General Hospital Tešanj); A Tursunovic (Tuzla; University Clinical Center Tuzla).

Bulgaria: T Ivanov (Pleven; Heart and Brain - Pleven Hospital); M Karamanliev (Pleven; University Hospital Dr Georgi Stranski, Medical University - Pleven); R Donchev (Plovdiv; MHAT St. Karidad); D Hadzhiev (Plovdiv; UMHAT Sveti Georgi); T Yotsov (Ruse; University Hospital Medika); E Hristova (Sofia; Fifth City Hospital Sofia - 5th MBAL).

Croatia: J Mihanovic (Zadar; Zadar General Hospital); G Augustin (Zagreb; University Hospital Centre Zagreb).

Cyprus**:** A Yiallourou (Nicosia; Nicosia General Hospital).

Czech Republic: J Moravik (Decin; Krajská zdravotní as - Hospital Decin); A Al Kaddah (Hradec Kralove; Charles University Hospital); Z Musilová (Ivančice; Hospital Ivančice); M Schön (Novy Jicin; Hospital & Oncological Centre Novy Jicin); J Roman (Ostrava; University Hospital Ostrava); B East (Prague; Motol University Hospital).

France: A Police (Eaubonne; Hôpital Simone Veil); A Castaldi (Nimes; Hôpital Carèmeau); E Reitano (Strasbourg; Nouvel Hopital Civil de Strasbourg).

Germany: C Kamphues (Berlin; Park-Klinik Weissensee); D Hackner (Erlangen; Universitätsklinikum Erlangen); U Ronellenfitsch (Halle; University Hospital Halle); J Rolinger (Moenchengladbach; Kliniken Maria Hilf); D Reim (Munich; Klinikum Rechts der Isar TUM School of Medicine); N Börner (Munich; Ludwig Maximilian University of Munich - Großhadern); J Goedeke (Munich; Ludwig Maximilian University of Munich - Innenstadt); AE Gut (München; Isarklinikum); M Janda (Rostock; University Hospital Rostock); J De Deken (Saarbruecken; Klinikum Saarbruecken); MW Löffler (Tuebingen; University Hospital Tuebingen).

Greece: K Kambouri (Alexandroupolis; Alexandroupolis University General Hospital); A Kyriakidis (Amfissa; General Hospital of Amfissa); D Korkolis (Athens; Agios Savvas Anticancer Hospital); N Memos (Athens; Aretaieion Hospital); D Kelgiorgi (Athens; Athens Euroclinic); C Chouliaras (Athens; Athens Medical Center); E Fradelos (Athens; Athens Naval and Veterans Hospital); N Michalopoulos (Athens; Attikon University General Hospital); N Dimitrokallis (Athens; Evaggelismos General Hospital); A Paspala (Athens; Evgenideio Hospital); P Christodoulou (Athens; General Hospital Asklepieio Voulas); EC Tampaki (Athens; KAT Athens General Hospital); D Schizas (Athens; Laiko University Hospital); K Kontzoglou (Athens; National and Kapodistrian University of Athens); M Spartalis (Athens; Sotiria General Hospital of Thoracic Diseases); M Billis (Filiates; General Hospital of Filiates); N Tsakiridis (Florina; Florina General Hospital ‘Eleni Th. Dimitriou’); A Karakosta (Ioannina; University Hospital of Ioannina); D Panagopoulos (Kyparissia; General Hospital of Messinia, Hospital Unit of Kyparissia); G Koukoulis (Larissa; General Hospital of Larissa ‘Koutlimpaneio and Triantafylleio’); G Christodoulidis (Larrisa; General University Hospital of Larissa); K Bouchagier (Patras; General University Hospital of Patras); V Mousafeiris (Patras; Karamandaneio Prefecture Children Hospital of Patras); A Papadopoulos (Piraeus; General Hospital of Nikaia); L Katsiaras (Piraeus; Metaxa Cancer Hospital); N Zampitis (Piraeus; Tzaneio General Hospital); O Ioannidis (Thessaloniki; George Papanikolaou General Hospital of Thessaloniki); M Drogouti (Thessaloniki; O Agios Dimitrios General Hospital); C Kaselas (Thessaloniki; Papageorgiou General Hospital); D Lytras (Volos; Achillopoyleio General Hospital of Volos).

Ireland: S Ramjit (Dublin; St James’s Hospital); R Tummon (Kerry; University Hospital Kerry).

Italy: R Sulce (Arezzo; Ospedale San Donato USL Toscana Sud Est); PM Cicerchia (Ariccia; Ospedale dei Castelli (N.O.C.)); E Marra (Aversa; San Giuseppe Moscati); M Rottoli (Bologna; IRCCS Azienda Ospedaliero-Universitaria di Bologna); J Andreuccetti (Brescia; ASST Spedali Civili, Ospedale di Brescia); E Locci (Cagliari; Cagliari University Hospital); F Cappellacci (Cagliari; Chirurgia Generale e Polispecialistica, Cagliari University Ospital ‘Duilio Casula’); N Cillara (Cagliari; Santissima Trinità - ATS Sardegna); E Abate (Carate Brianza (MB); Ospedale Vittorio Emanuele III - Carate Brianza); F Ascari (Carpi; Ramazzini); S Romano (Casarano; Francesco Ferrari Hospital); M Veroux (Catania; Azienda Ospedaliero- Universitaria Policlinico San Marco); B Nardo (Cosenza; Azienda Ospedaliera di Cosenza); D Sasia (Cuneo; Santa Croce e Carle Hospital, Cuneo); G Baronio (Esine; ASST Valcamonica Ospedale di Esine); N Fabbri (Ferrara; Azienda Unità Sanitaria Locale di Ferrara); J Martellucci (Firenze; Azienda Ospedaliera Universitaria Careggi); G Canonico (Firenze; Ospedale San Giovanni di Dio); V Lizzi (Foggia; Ospedali Riuniti Azienda Ospedaliera Universitaria); F D’acapito (Forlì; Morgagni-Pierantoni); D Merlini (Garbagnate Milanese; ASST Rhodense - Ospedale di Garbagnate Milanese); A Barberis (Genoa; E.O. Ospedali Galliera); MF Amisano (Genoa; IRCCS Ospedale Policlinico San Martino); A Luzzi (Genoa; Ospedale Villa Scassi); F Palmieri (Gravedona ed Uniti; Ospedale Moriggia Pelascini); CL Bertoglio (Magenta; Ospedale ‘G.Fornaroli’, ASST-OVEST Milanese); E Baldini (Melzo; Ospedale Santa Maria delle Stelle, ASST Melegnano Martesana); M Ceolin (Milan; Humanitas Research Hospital); P De Nardi (Milan; IRCCS San Raffaele Scientific Institute, Milan); MG Piacentini (Milan; Ospedale Fatebenefratelli e Oftalmico); F Ferrara (Milan; San Carlo Borromeo); F Brucchi (Milan; Sesto San Giovanni Hospital); F Di Marco (Modica; Ospedale Maggiore); N Tamini (Monza; Fondazione IRCCS San Gerardo dei Tintori Monza, Scuola di Medicina e Chirurgia, Università Milano Bicocca); P Anoldo (Naples; Federico II University of Naples); R Patrone (Naples; Istituto Nazionale Tumori Fondazione, Pascale IRCCS); F Selvaggi (Naples; Primo Policlinico di Napoli); G Bellio (Padova; Piove di Sacco Hospital); P Venturelli (Palermo; Policlinico Universitario Paolo Giaccone); L Conti (Piacenza; G. Da Saliceto); L Morelli (Pisa; Azienda Ospedaliero Universitaria Pisana); SMM Basso (Pordenone; Azienda Sanitaria Friuli Occidentale (AS FO)); F Biolchini (Reggio Emilia; Azienda Unità Sanitaria Locale - IRCCS di Reggio Emilia); C Marafante (Rivoli; Ospedale degli Infermi di Rivoli); A Antinori (Rome; Fondazione Policlinico Universitario Agostino Gemelli); M Campanelli (Rome; Policlinico Tor Vergata Hospital, Rome); P Lapolla (Rome; Policlinico Umberto I Sapienza University of Rome); G Palomba (Salerno; San Giovanni di Dio e Ruggi d’Aragona); L Cardinali (San Benedetto del Tronto; Madonna del Soccorso Hospital); E Andolfi (Sansepolcro; Valtiberina); L Verre (Siena; Azienda Ospedaliero Universitaria Senese); G Poillucci (Spoleto (PG); San Matteo degli Infermi); E Pontecorvi (Sulmona; SS Annunziata); S Novello (Treviso; Ospedale Ca’ Foncello - Università di Padova (DISCOG)); M Santarelli (Turin; Città della Salute e della Scienza); L Cobellis (Vallo Della Lucania; Casa di Cura Prof. Dott. Luigi Cobellis); G Ietto (Varese Lombardy; University of Insubria, Ospedale di Circolo e Fondazione Macchi (Varese)); F Pederiva (Varese; Filippo Del Ponte Hospital, University of Insubria); A Iacomino (Venezia; Ospedale Civile - Santi Giovanni e Paolo); D Verdi (Venice; Mirano Hospital); A Broglia (Voghera; Ospedale Civile di Voghera); P Cianci (andria; Lorenzo Bonomo); M Angelucci (rome; policlinico universitario campus bio medico of rome); G Calini (udine; santa maria della misericordia).

Lithuania: D Venskutonis (Kaunas; LUHS Kaunas Hospital); A Dauksa (Kaunas; Lithuanian University of Health Sciences Kaunas Clinics); A Gulla (Vilnius; Vilnius University Hospital).

Malta: J Psaila (Msida; Mater Dei Hospital).

Republic of North Macedonia: T Risteski (Skopje; University Clinic for Pediatric Surgery).

Poland: Ł Nawacki (Kielce; Wojewódzki Szpital Zespolony w Kielcach); M Kisielewski (Krakow; 5th Military Clinical Hospital); Z Orzeszko (Krakow; Brothers Hospitallers Hospital); M Matyja (Krakow; Jagiellonian University Medical College); W Krawczyk (Sosnowiec; Wojewódzki Szpiital Specjalistyczny nr 5 im. Św Barbary); M Walędziak (Warsaw; Military Institute Of Medicine); JK Zajac (Wroclaw; Regional Specialist Hospital in Wroclaw); F Brzeszczyński (Łódź; Copernicus Memorial Hospital).

Portugal: S Henriques (Almada; Hospital Garcia de Orta); J Frazão (Amadora; Hospital Prof. Doutor Fernando Fonseca, E.P.E.); S Gaspar Reis (Barreiro; Centro Hospitalar Barreiro Montijo, EPE); AR Mateus Loureiro (Caldas da Rainha; Hospital das Caldas da Rainha - Centro Hospitalar do Oeste, E.P.E); M Reia (Elvas; Hospital Santa Luzia Elvas); J Pinho (Figueira da Foz; Hospital Distrital da Figueira da Foz); DG Alves (Funchal; Hospital Dr. Nélio Mendonça); R Silva Borges (Horta; Hospital da Horta, E.P.E.); E Borges (Lisbon; Centro Hospitalar Lisboa Norte); M Nunes (Matosinhos; Unidade Local de Saude de Matosinhos - Hospital Pedro Hispano); A Faustino (Ponta Delgada; Hospital do Divino Espírito Santo); G Fialho (Portalegre; Hospital Doutor José Maria Grande); J Dias-Ferreira (Porto; Centro Hospitalar e Universitário de São João); M Santos (Porto; Hospital da Prelada); J Marques Antunes (Santa Maria da Feira; Centro Hospitalar Entre o Douro e Vouga); H Devesa (Santarem; Hospital de Santarem); J Ricardo (Santiago do Cacém; Hospital do Litoral Alentejano); R Branquinho (Tomar; Centro Hospitalar Médio Tejo); J Fernandes (Vila Franca de Xira; Hospital Vila Franca de Xira); J Pereira-Macedo (Vila Nova de Famalicao; Centro Hospitalar do Medio Ave); B Vieira (Vila Real; Centro Hospitalar de Trás-os-Montes e Alto Douro, E.P.E.).

Romania: ST Makkai-Popa (Brasov; Regina Maria); F Grama (Bucharest; Coltea Clinical Hospital); EA Toma (Bucharest; Elias Emergency Hospital); I Negoi (Bucharest; Emergency Clinical Hospital Bucharest); M Muresan (Cluj-Napoca; Medicover Hospital Cluj).

Russian Federation: V Kakotkin (Kaliningrad; Immanuel Kant Baltic Federal University, Regional Clinical Hospital); A Bedzhanyan (Moscow; Petrovsky National Research Centre of Surgery); S Katorkin (Samara; Hospital Surgery Clinic of Samara State Medical University); A Butyrskii (Simferopol; Municipal Emegency Hospital No.6); V Ten (Yuzhno-Sakhalinsk; Private healthcare institution ‘RZD-Medicine’).

Serbia: A Karamarkovic (Belgrade; Zvezdara University Medical Center).

Slovenia: JA Košir (Ljubljana; University Medical Centre).

Spain: H Aguado López (Albacete; Hellín Hospital); MDM Martí-Ejarque (BARCELONA; Hospital Universitari Sagrat Cor); A Torroella (Barcelona; HM Nou Delfos); O Martin Sole (Barcelona; Hospital Sant Joan de Deu); A Landaluce-Olavarria (Bizkaia; Hospital Urduliz); V Alonso (Burgos; Hospital Universitario de Burgos); Á Fernández Camuñas (Ciudad Real; Hospital General Universitario de Ciudad Real); M Estaire Gómez (Leganés; Severo Ochoa University Hospital); AG Barranquero (Lleida; Hospital Universitari Arnau de Vilanova); L Marquez (Madrid; Hospital Central de la Cruz Roja San Jose y Santa Adela); P Serrano Méndez (Madrid; Hospital Clinico San Carlos); A Vilar (Madrid; Hospital Universitario Principe de Asturias); J Guevara (Madrid; Hospital Universitario la Paz); AM Minaya Bravo (Madrid; Hospital del Henares); JL Rodicio Miravalles (Oviedo; Hospital Universitario Central de Asturias (HUCA)); R Díaz Pedrero (Rivas-Vaciamadrid, Madrid; Hospital Universitario HM Rivas); L Tallon-Aguilar (Sevilla; Hospital Universitario Virgen del Rocio); A Curado Soriano (Seville; Hospital Universitario Virgen Macarena); Z Balciscueta (Valencia; Hospital Arnau de Vilanova); D Moro-Valdezate (Valencia; Hospital Clínico Universitario de Valencia); B De Andrés-Asenjo (Valladolid; Hospital Clínico Universitario de Valladolid); A Vazquez Melero (Vitoria-Gasteiz; Hospital Universitario Araba); J Escartin (Zaragoza; Hospital Royo Villanova); C Gracia-Roche (Zaragoza; Hospital Universitario Miguel Servet).

Sweden: H Zaigham (Malmö; Skåne University Hospital); M Nikberg (Vasteras; Västmanlands Hospital Västerås).

Switzerland: P Probst (Frauenfeld; Spital Thurgau AG); E Gialamas (Geneva; Geneva University Hospitals); J Gass (Luzern; Luzerner Kantonsspital); A Tampakis (Olten; Kantonsspital Olten); G Peros (Winterthur; Kantonsspital Winterthur); MA Schneider (Zurich; University Hospital of Zurich).

United Kingdom: CS Ong (Bangor, North Wales; Ysbyty Gwynedd); P Patel (Barrow in Furness; Furness General Hospital); S Dindyal (Basildon; Basildon University Hospital); F Georgiades (Bedford; Bedford Hospital); J Abbasy (Birmingham; Heartlands Hospital); M Kaur (Brighton; Royal Sussex County Hospital); B Martin (Bristol; Bristol Royal Hospital for Children); M Chauhan (Camberley; Frimley Health NHS FT - Frimley Park); S Ahmed (Chester; Countess of Chester Hospital); M Tutton (Colchester; Colchester Hospital University); N Chidumije (Coventry; University Hospitals Coventry and Warwickshire NHS Trust); W Al-Khyatt (Derby; Royal Derby Hospital); A Sukumar (Dudley; Russell’s Hall Hospital); H Kamal (Dundee; Ninewells Hospital); A Nada (Durham; University Hospital North Durham); A Chaudhary (Exeter; Royal Devon and Exeter Hospital); M Bogdan (Great Yarmouth; James Paget Univeristy NHS Foundation Trust Hospital); M Peter (Huddersfield; Huddersfield Royal Infirmary); J Walshaw (Hull; Hull University Teaching Hospitals NHS Trust); M Ewedah (Ilford; King George Hospital); L Rampersad (Larbert; Forth Valley Royal Hospital); A Peckham-Cooper, NS Blencowe (Leeds; Leeds Teaching Hospitals); R Lunevicius (Liverpool; Aintree University Hospital); P Panahi (London; Ealing Hospital); E Baili (London; Guy’s and St Thomas’ Hospitals); K Theodoropoulou (London; Homerton University Hospital); P Kapsampelis (London; Kingston); MMH Mohammed (London; Queen Elizabeth Hospital, Woolwich); C Parmar (London; The Whittington Hospital); MMH Mohammed (London; University Hospital Lewisham); C Smart (Macclesfield; Macclesfield District General Hospital); P Wilson (Manchester; Wythenshawe Hospital); F Gareb (Margate; Queen Elizabeth the Queen Mother Hospital Margate); G Sundaram Venkatesan (Middlesbrough; James Cook University Hospital); C Hidalgo Salinas (Morecambe; Royal Lancaster Infirmary); S Tingle (North Shields; Northumbria NHS Hospital Trust); N Marzouqa (Nottingham; Queens Medical Centre); T Theivendrampillai (Oxford; John Radcliffe Hospital); M Zahed Abdalla (Portsmouth; Queen Alexandra Hospital); A Rahman (Redhill; East Surrey Hospital); M Abdelkarim (Rhyl; Glan Clwyd Hospital); O Whitehurst (Salford; Salford Royal Hospital); E Tokidis (Sheffield; Sheffield Teaching Hospital NHS Foundation Trust); S Bandyopadhyay (Southampton; Southampton General Hospital); A Evans (Swansea; Morriston Hospital Swansea); A Salam (Walsall; Walsall Manor Hospital).

**Collaborators** (listed by country and city)**:**

Albania: B Ibi, S Faber (Korca; Regional Hospital of Korca); I Dajti (Tirana; University hospital Koco Gliozheni).

Austria: N Klammer, R Schmidt-Branden, P Tschann, P Horvath (Feldkirch; Landeskrankenhaus Feldkirch); N Koter, G Moitzi, E Wallner, C Allmer, F Aigner (Graz; Barmherzige Brüder Krankenhaus, Graz); J Kahn, A Belarmino, R Sucher, V Wolfschluckner, G Singer (Graz; Medical University of Graz); R Függer, M Biebl, A Punzengruber, H Fehrer (Linz; Ordensklinikum Linz Elisabethinen); A Binder, E Haiden, P Riedl, M Enßlin (Tulln; Universitätsklinikum Tulln).

Bosnia and Herzegovina: M Stjepanovic (Doboj; Genera Hospital ‘Sveti aposto Luka’ Doboj); O Čančar, M Pejović (Foča; University Hospital Foča); J Miskovic, M Boras, M Kajic, V Dragisic, Z Brekalo, I Mikulic, N Soldo, M Bevanda, M Faletar (Mostar; SKB University Clinical Hospital Mostar); M Salibašić, E Hodžić, E Halilović, M Kruščica (Sarajevo; Clinical Center University of Sarajevo); A Cerovac, H Škiljo, E Hodžić, O Bedak, M Kalabić, E Begunić (Tešanj; General Hospital Tešanj); A Huremovic, E Alić (Tuzla; University Clinical Center Tuzla).

Bulgaria: D Georgiev, I Fidoshev, V Neykov, E Daleva, I Ilieva (Pleven; Heart and Brain - Pleven Hospital); M Karamanliev, D Dimitrov, A Shanker, P Vladova, MD Shoshkova, A Mehta, M Abdullahi, V Ratheesh, V Kamalathevan, C Wiesner, S Shittu, M Galasyuk, S Shanker (Pleven; University Hospital Dr Georgi Stranski, Medical University - Pleven); M Imirski, A Soumpasis (Plovdiv; MHAT St. Karidad); E Hadzhieva, D Chakarov (Plovdiv; UMHAT Sveti Georgi); T Yotsov, P Kamenova, A Vricheva, I Yotsov (Ruse; University Hospital Medika); E Hristova, K Spassov (Sofia; Fifth City Hospital Sofia - 5th MBAL).

Croatia: J Mihanovic, I Bacic, D Vukosav , V Žufić, O Jurić, E Dijan, N Jović, I Ćoza, I Rakvin, Z Katusic, T Soric, I Vidić, D Rukavina (Zadar; Zadar General Hospital); I Separovic, R Radojković, J Mavrek (Zagreb; University Hospital Centre Zagreb).

Czech Republic: T Reichelt, P Hudáč (Decin; Krajská zdravotní as - Hospital Decin); K Akter, L Moolla, F Rudisch, A Ibrahim Hassan, O Ahmad, E ELShennawy, M Shalaby, M Khaled, A Akiba, F Philips, E Bankart, R Elshennawy, A Al Kaddah, H Al Atassi, S Ashry, N Salgadoe (Hradec Kralove; Charles University Hospital); L Majerčák , P Levíček, A Lukáč, L Pánči (Ivančice; Hospital Ivančice); J Roman, L Tulinsky, I Mrazkova, P Ostruszka, A Varga, L Martinek (Ostrava; University Hospital Ostrava); H Novák, J Woleský, P Francúz (Prague; Motol University Hospital).

France: E Volpin, H Braham, C Lionel, R Arena, Y Malki (Eaubonne; Hôpital Simone Veil); M Bertrand, A Castaldi, L Theuil, A L’Hostis, M Prudhomme (Nimes; Hôpital Carèmeau); P Riva, A Lapergola, D Mutter, S Perretta (Strasbourg; Nouvel Hopital Civil de Strasbourg).

Germany: LD Lee (Berlin; Park-Klinik Weissensee); J Binder, A Denz, C Krautz, M Brunner, M Maak, GF Weber, A Stollberg, D Hackner, S Engel, F Krämer, R Grützmann (Erlangen; Universitätsklinikum Erlangen); M Schüler, J Kleeff, R Rüdrich (Halle; University Hospital Halle); A Kirschniak, J Rolinger, S Göller, L Van den Hil , J Miller, H Pehlivan (Moenchengladbach; Kliniken Maria Hilf); M Kießler, N Hüser, D Schippers, M Berlet, M Steffani, M Weber (Munich; Klinikum Rechts der Isar TUM School of Medicine); N Börner, M Albertsmeier, H Arbogast, M Mattis, S Jarmusch, P Zimmermann, U Wirth (Munich; Ludwig Maximilian University of Munich - Großhadern); F Anzinger (Munich; Ludwig Maximilian University of Munich - Innenstadt); FG Bader, M Sohn, ML Koschke, M Busch , N Hielscher (München; Isarklinikum); A Brosin, J Lindert, M Philipp, M Gumsheimer, F Wiese (Rostock; University Hospital Rostock); L Sahan, GA Stavrou, J De Deken, M Jabal (Saarbruecken; Klinikum Saarbruecken); MW Löffler, A Königsrainer, M Quante, C Yurttas (Tuebingen; University Hospital Tuebingen).

Greece: I Gogoulis, K Bekiaridou, A Mitsala, S Botaitis, C Tsalikidis, M Asimakidou, C Nikolaou, E Efremidou, C Limas, P Chloropoulou, M Aggelidou, M Pitiakoudis, P Kostoglou, G Pappas Gogos, M Karanikas (Alexandroupolis; Alexandroupolis University General Hospital); E Kapasakis, M Karakeke, A Skarpas, C Floros, K Athanassiou, E Karakeke (Amfissa; General Hospital of Amfissa); N Tasis, A Sarafi, A Plastiras, G Kavalieratos, T Tsirlis (Athens; Agios Savvas Anticancer Hospital); L Chardalias, N Memos, V Themelidi, I Papaconstantinou, T Theodosopoulos, D Politis, K Iliakopoulos, K Bramis, P Antonakis, A Skreka, D Kotsaris, N Dafnios, A Vezakis, I Contis, T Petropoulou, KC Kordeni, T Kozonis, G Fragulidis, D Massaras (Athens; Aretaieion Hospital); E Apostolopoulos, I Karatsolis, A Mourtzouni, K Avgerinos, D Kelgiorgi, K Polychronopoulos, G Kostoulas, A Tsechpenakis, A Saridaki (Athens; Athens Euroclinic); A Ioannidis, C Chouliaras, I Tierris, MK Konstantinidis (Athens; Athens Medical Center); DK Manatakis, D Balalis, N Stamos, N Tasis, V Kalles (Athens; Athens Naval and Veterans Hospital); T Sidiropoulos, M Papadoliopoulou, N Arkadopoulos, D Sampanis, P Vassiliu, E Dylja, AI Nikolaou, I Margaris, P Kokoropoulos, V Tsaousis, S Christodoulou, E Poulios, A Chamzin (Athens; Attikon University General Hospital); S Kapiris, E Mavrodimitraki, M Sotiropoulou, N Dimitrokallis, N Papadogianni, V Vougas, M Papamichail, K Rekouna, K Pavlopoulos, N Roukounakis, A Thanasa, P Trakosari, M Christou, E Saitoglou (Athens; Evaggelismos General Hospital); C Nastos, D Dellaportas, P Lykoudis, N Garmpis, G Kouraklis (Athens; Evgenideio Hospital); P Christodoulou, G Kapogiannatos, A Nikitaras, SM Tsoti, J Katogiritis (Athens; General Hospital Asklepieio Voulas); EC Tampaki, C Papazacharias, O Bellou (Athens; KAT Athens General Hospital); N Machairas, P Dorovinis, C Doudakmanis, D Schizas, A Syllaios, MD Keramida, P Stamopoulos, S Davakis, M Despotidis, A Panagakis, S Kykalos, F Stavratis, M Vailas, L Karydakis, N Kydonakis, A Loizou, KS Giannakopoulos, P Sakarellos, A Kozadinos, I Katsaros (Athens; Laiko University Hospital); C Damaskos, E Antoniou, M Mavri, I Psilopatis, S Vernadakis , I Bokos, P Paraskeva, D Vardakostas, I Gomatos, A Barlas, A Smyrnis, D Prevezanos, NN Mathioudakis, I Kozadinos, A Kozadinos, P Kanavidis, N Garmpis (Athens; National and Kapodistrian University of Athens); E Spartalis, M Spartalis (Athens; Sotiria General Hospital of Thoracic Diseases); C Stefanou, S Gkogkos, L Fountoulis, I Sougkas, M Billis, A Kontokostopoulos, A Balta, T Padioti , MA Sotiriou, A Theochari (Filiates; General Hospital of Filiates); N Tsakiridis, I Tsakiridis, E Synekidou (Florina; Florina General Hospital ‘Eleni Th. Dimitriou’); E Athanasopoulou, C Ntagkas, E Samara, A Katsiou (Ioannina; University Hospital of Ioannina); D Panagopoulos, A Panagopoulos, L Katsiaras (Kyparissia; General Hospital of Messinia, Hospital Unit of Kyparissia); C Kolla, L Mansour, G Koukoulis, S Zourntou, D Papageorgouli, K Bouliaris, LI Fountarlis, M Bei, AA Kalidis, X Vagena, A Gkouniaroudi, A Migdanis, A Bakalis, E Gavriil (Larissa; General Hospital of Larissa ‘Koutlimpaneio and Triantafylleio’); K Koumarelas, MN Kouliou (Larrisa; General University Hospital of Larissa); K Bouchagier, F Mulita, G Verras, G Skroubis, I Maroulis (Patras; General University Hospital of Patras); V Mousafeiris, A Panagidis (Patras; Karamandaneio Prefecture Children Hospital of Patras); A Papadopoulos, P Grivas, F Spanos, A Kalogeropoulou , G Zeringa, C Kourouniotis, G Rados, E Barkolias , K Zakkas, I Demiris, V Nikolaou, K Tata, G Karakaidos (Piraeus; General Hospital of Nikaia); E Kontis, E Papamattheou, A Efstathiou, E Efstathiou, N Kopanakis, K Ntatsis, I Katsaros, P Manikis, V Tselepidis, M Kyriazi (Piraeus; Metaxa Cancer Hospital); I Siannis, N Kouzakos, V Georgilaki , N Vlachakos, S Vederaki, A Tsiaka, A Zarafidou, N Zampitis, S Tsatsos, F Stefou, F Kyramargios, M Merrakos, G Bekakos, A Marinis (Piraeus; Tzaneio General Hospital); O Ioannidis, E Anestiadou, K Zapsalis, S Simeonidis, S Bitsianis (Thessaloniki; George Papanikolaou General Hospital of Thessaloniki); M Drogouti, A Gkoutoula, A Sarakatsanos, E Efthymiou, I Chatzis (Thessaloniki; O Agios Dimitrios General Hospital); I Spyridakis, C Kaselas, M Tsopozidi, M Florou, C Demiri, V Papadopoulos, D Giakoustidis, A Giakoustidis, D Alexandrou, P Chatzikomnitsa (Thessaloniki; Papageorgiou General Hospital); SC Liapis, K Perivoliotis, C Chatzinikolaou, N Tsantikos, ZR Karampotaki , D Lytras (Volos; Achillopoyleio General Hospital of Volos).

Italy: M De Prizio, K Kröning, R Sulce, LM Fatucchi, F Tofani, V Mariottini, R Malatesti, M Scricciolo, GA Pellicano’, A D’Ignazio, A Mazzoni, A Biancafarina, M Angelini, V Borgogni (Arezzo; Ospedale San Donato USL Toscana Sud Est); L Rossi, G Munzi, G Tarantino, M Castrovillari, A Serao , JR Casella Mariolo, G Del Corpo, A Natili, A Iodice (Ariccia; Ospedale dei Castelli (N.O.C.)); S Gargiulo, B Esposito, M Pannullo, L Bracciano, E Marra, A Alberico (Aversa; San Giuseppe Moscati); A Gori, S Cardelli, G Dajti, C Larotonda, IS Russo (Bologna; IRCCS Azienda Ospedaliero-Universitaria di Bologna); J Andreuccetti, S Molfino, D Alberti, G Pignata, G Emiliani, G Boroni, M Ruffoli, M Manfredini, L Sequi, G Zanni (Brescia; ASST Spedali Civili, Ospedale di Brescia); E Locci, M Podda, V Murzi, C Piras , A Carta, A Pisanu, T Pilia, P Marongiu, A Saba, M Pisano, F Campus, E Gessa, E Silanos, A Lai, F Frongia, F Corronca , S Montisci (Cagliari; Cagliari University Hospital); F Cappellacci, C Soddu, GL Canu, F Medas, PG Calò, S Puddu, M Biancu, M Abbas, F Casti (Cagliari; Chirurgia Generale e Polispecialistica, Cagliari University Ospital ‘Duilio Casula’); B Demurtas, A Deserra, F D’Agostino, C Margiani (Cagliari; Santissima Trinità - ATS Sardegna); L Laface, M Casati, M Mariani, S Guarriello, A Balconi (Carate Brianza (MB); Ospedale Vittorio Emanuele III - Carate Brianza); B Scotto, N Laquatra, R Ruccella, G De Angeli (Carpi; Ramazzini); G Gravante, A Chiappini , R Lopatriello, G Mammolo (Casarano; Francesco Ferrari Hospital); C Distefano, G Riccioli, R Granata, M Veroux, DC Centonze, S Costa, R Gioco, D Zerbo, A Licciardello, L Stella (Catania; Azienda Ospedaliero- Universitaria Policlinico San Marco); L Rende, M Osso, D Paglione, F Pata (Cosenza; Azienda Ospedaliera di Cosenza); D Sasia, G Giraudo, D Ribero, S Alberti, V Schirinzi (Cuneo; Santa Croce e Carle Hospital, Cuneo); P Belotti, L Taglietti (Esine; ASST Valcamonica Ospedale di Esine); V Giordano, A Pesce, CV Feo, MC Pignanelli, S Severi (Ferrara; Azienda Unità Sanitaria Locale di Ferrara); F Cammelli, F Natali, M Scheiterle, G Maltinti, L Fortuna, F Coratti, A Manetti , J Martellucci, E Monati (Firenze; Azienda Ospedaliera Universitaria Careggi); L Gabellini, R Fratarcangeli, A Damigella, E Adinolfi, A Anastasi (Firenze; Ospedale San Giovanni di Dio); M Montagna, A Giuliani, G Procaccini, N Tartaglia, F Vovola, D Merlicco, S Schirone , ST Massa, G Pavone, M Pacilli, F Maffei, A Gerundo (Foggia; Ospedali Riuniti Azienda Ospedaliera Universitaria); D Di Pietrantonio, S Quartarone , L Solaini, G Ercolani, L Ragazzini, V Zucchini (Forlì; Morgagni-Pierantoni); M Cammelli, D Scotto Di Carlo, FAN Marin (Garbagnate Milanese; ASST Rhodense - Ospedale di Garbagnate Milanese); A Azzinnaro, A Razzore, A Petrungaro, E Mina, B Sperotto (Genoa; E.O. Ospedali Galliera); G Carganico, D Pertile, D Soriero (Genoa; IRCCS Ospedale Policlinico San Martino); R Diaz, A Luzzi, S Carrabetta, C Meola, D Caruso, F Floris, P Grondona, E Romairone, S Marzorati, F Ré, C Righetti, A Viacava, L Epis (Genoa; Ospedale Villa Scassi); R Sampietro, D Gobatti, C Zandonella (Gravedona ed Uniti; Ospedale Moriggia Pelascini); VP Dinuzzi, U Rivolta, S Luciano, GMF Marini, L Scaravilli (Magenta; Ospedale ‘G.Fornaroli’, ASST-OVEST Milanese); R Magarini, G Saletta, AC Sironi, G Grava, M Mercurio (Melzo; Ospedale Santa Maria delle Stelle, ASST Melegnano Martesana); D Zulian, A Izzo, M Gritti, S Giudici, E Desiato (Milan; Humanitas Research Hospital); M Molteni, L Ottaviani (Milan; IRCCS San Raffaele Scientific Institute, Milan); G Grande, E Mazzotta (Milan; Ospedale Fatebenefratelli e Oftalmico); S Grimaldi (Milan; San Carlo Borromeo); F Brucchi, F Ferraina, S Lauricella (Milan; Sesto San Giovanni Hospital); E Di Marco (Modica; Ospedale Maggiore); LC Nespoli, G De Carlo, EA Baccalini, D Palmisano, A Scacchi, N Tamini, L Ripamonti, M Rennis, C Vitiello, A Davolio, L Degrate, P Masseria, V Brocco, P Chiacchio, M Ceresoli, E Signaroli, A Finocchio, C Fumagalli, M Binda (Monza; Fondazione IRCCS San Gerardo dei Tintori Monza, Scuola di Medicina e Chirurgia, Università Milano Bicocca); M Milone, M Manigrasso, S Vertaldi, A D’Amore, GD De Palma, A Marello, L Fedele, C Sorrentino, D Pignatelli , G Luglio, FP Tropeano, M Cricrì, A Miele, G Aprea, G Palomba, M Capuano, R Basile, G Sorrentino (Naples; Federico II University of Naples); D Rega, A Ottaiano, V Granata, A Belli, F Izzo (Naples; Istituto Nazionale Tumori Fondazione, Pascale IRCCS); G Pellino, D Massaro, V Mosca, F Selvaggi (Naples; Primo Policlinico di Napoli); G Bellio, L Rubin, N De Santis, N Schiavon, A Zerbinati, S Corso, C Cecconi (Padova; Piove di Sacco Hospital); P Venturelli, G Cocorullo, G Carollo, R Tutino, A Bonelli, G Salamone, MP Proclamà, R Guercio, L Licari, N Finocchiaro, G Graziano, G Orlando, G Guercio, M Marcianò, G Galatioto, F Vassallo (Palermo; Policlinico Universitario Paolo Giaccone); G Palmieri, F Banchini (Piacenza; G. Da Saliceto); G Di Franco, F Porcelli, N Furbetta, A Comandatore, S Guadagni, M Palmeri (Pisa; Azienda Ospedaliero Universitaria Pisana); P Ubiali, F Maffeis, J Velkoski (Pordenone; Azienda Sanitaria Friuli Occidentale (AS FO)); M Giuffrida, GE Nita (Reggio Emilia; Azienda Unità Sanitaria Locale - IRCCS di Reggio Emilia); C Marafante, M Garino, SL Birolo , M Dugo, M Pisano, A Borello, R Barone, LD Bonomo, MV Facchino, M Caccetta, E Moggia, MR D’Anna, C Mosca, S Mungo, A Masciandaro (Rivoli; Ospedale degli Infermi di Rivoli); F Tirelli, I Neri , M Aulicino, C Vacca (Rome; Fondazione Policlinico Universitario Agostino Gemelli); M Campanelli, M Grande, L Siragusa, G Sica (Rome; Policlinico Tor Vergata Hospital, Rome); A Mingoli, G Brachini, B Cirillo, S Meneghini, S Giovampietro , G Duranti, F Ciccarone, L Simonelli, I Clementi, B Binda, GB Fonsi, E Spalice , E Cianci, MI Bellini, G Sgarzini, S Sorrenti, E Lori, P Palumbo, D Pironi (Rome; Policlinico Umberto I Sapienza University of Rome); A Amendola, C De Martino, E Bisogno (Salerno; San Giovanni di Dio e Ruggi d’Aragona); S Di Saverio, A Morello, L Lely, I Merlini, G Travaglini , S Sabbatini, M Zambon (San Benedetto del Tronto; Madonna del Soccorso Hospital); D Giulitti, L Barni, GE Poto (Sansepolcro; Valtiberina); D Fusario, L Resca, L Carbone, A Francia, GE Poto, AL Pesce, O Carpineto Samorani, F Roviello, A Ongaro, SA Piccioni, M Gambelli, L Catozzi, M Gjoka, A Bartalini Cinughi de Pazzi, NN Leonelle Lore, G Grassi, F Manasci, V Ricchiuti (Siena; Azienda Ospedaliero Universitaria Senese); E Basile, A Spaziani (Spoleto (PG); San Matteo degli Infermi); V Silvestri, M Favoriti, P Favoriti (Sulmona; SS Annunziata); S Novello, M Piccino, R Baldan, U Grossi, G Zanus, F Scolari, E De Leo, M Brizzolari, A Brun-Peressut, I Hoxhaj, M Scopelliti (Treviso; Ospedale Ca’ Foncello - Università di Padova (DISCOG)); LB Lo Piccolo, E Montanari, D Cianflocca, A Marano, S Galati, SL Gamba, F Velluti, A Caltagirone, M Giuliano , E Potenza, B De Zolt Ponte, D Visconti, VU De Donato, L Capello, S Chaifouroosh Mamagany, C Celano (Turin; Città della Salute e della Scienza); E Donnarumma, C Saviello, R Scola (Vallo Della Lucania; Casa di Cura Prof. Dott. Luigi Cobellis); S Megna, M Berselli, N Palamara, L Liepa, E Ferri (Varese Lombardy; University of Insubria, Ospedale di Circolo e Fondazione Macchi (Varese)); V Gentilino, M Mogiatti, G Farris, N Pasqua (Varese; Filippo Del Ponte Hospital, University of Insubria); A Iacomino (Venezia; Ospedale Civile - Santi Giovanni e Paolo); I Mondi, C Da Lio, F Sulo, E Ciccioli, D Verdi (Venice; Mirano Hospital); M Martorana, M Filardo, L Schiavone (Voghera; Ospedale Civile di Voghera); I Conversano, M Cappiello, G Scialandrone, N Petrarota, R Tumolo (andria; Lorenzo Bonomo); M Angelucci, S Valeri, G Pascarella, A Strumia, R Alloni (rome; policlinico universitario campus bio medico of rome); D Muschitiello, V Morinelli, L Bonello, SG Intini, S Moschella (udine; santa maria della misericordia).

Lithuania: E Dainius, S Bradulskis, E Margelis, A Mačiulaitytė, A Subocius, A Parseliunas, E Kubiliute, D Zuikyte, J Kutkevičius, J Vaitekūnas (Kaunas; LUHS Kaunas Hospital); L Venclauskas, K Jasaitis, M Jokubauskas, Z Dauksa (Kaunas; Lithuanian University of Health Sciences Kaunas Clinics); A Gulla, E Daukšaitė (Vilnius; Vilnius University Hospital).

Malta: C Cini, A Sultana, M Farrugia, J Schembri Higgans, S Bowman, J Psaila, P Andrejevic, S Brincat, K Muscat, M Sammut, R Abela, M Zammit Vincenti, L Casingena, J Galea, M Portelli, M Sammut, N Spiteri, K Iles, R Cachia, D Hili (Msida; Mater Dei Hospital).

Republic of North Macedonia: T Risteski, V Naunova, L Jovcheski (Skopje; University Clinic for Pediatric Surgery).

Poland: R Mazurkiewicz, M Kołomańska, PJ Milewski (Kielce; Wojewódzki Szpital Zespolony w Kielcach); M Kisielewski, T Stefura, K Richter, W Wysocki, N Kłos , W Jabłoński, I Alsoubie, B Żaczek, T Wojewoda, J Bolanowski (Krakow; 5th Military Clinical Hospital); Z Orzeszko, M Wikar, R Solecki, B Markowska, M Szura, T Gach (Krakow; Brothers Hospitallers Hospital); M Matyja, B Habrat, W Serednicki (Krakow; Jagiellonian University Medical College); Z Lorenc, M Święch, M Mietła, W Krawczyk, M Nycz (Sosnowiec; Wojewódzki Szpiital Specjalistyczny nr 5 im. Św Barbary); K Urbańska, K Komorowska, P Kowalewski, M Walędziak (Warsaw; Military Institute Of Medicine); JK Zajac, M Zawadzki (Wroclaw; Regional Specialist Hospital in Wroclaw); M Kusiński, M Pryt, F Brzeszczyński , H Dąbrowski, M Redynk (Łódź; Copernicus Memorial Hospital).

Portugal: J Figueiredo, B Cismasiu, R Souto, S Henriques, AL Preto Barreira, J Vaz, JM Carlos, M Trindade, L Moreira, M Palas, J Simoes (Almada; Hospital Garcia de Orta); F Ramalho de Almeida, M Vasconcelos, A Neves, J Ribeiro, F Afonso , A Pita, R Miranda Pera, M Bernardo, C Rio Ferreira, T Branco, J Fontaínhas, S Pimentel Morais, B Pinto (Amadora; Hospital Prof. Doutor Fernando Fonseca, E.P.E.); S Patrocínio, L Moniz, C Rolo Santos, P Bernardo, F Nazareth (Barreiro; Centro Hospitalar Barreiro Montijo, EPE); C Silva, L Heeren, AR Mateus Loureiro, B Tinoco, A Abreu (Caldas da Rainha; Hospital das Caldas da Rainha - Centro Hospitalar do Oeste, E.P.E); DM Gonçalves Múrias Gomes, C Figueiredo, C Aguero, M Reia, M Guerrero, MA Fernandez Romero, J Dominguez (Elvas; Hospital Santa Luzia Elvas); I Colaço, M Nunes Luís, S Andrade, S Oliveira, D Pais (Figueira da Foz; Hospital Distrital da Figueira da Foz); DG Alves, F Castro, R Ribeiro, I Mogárrio, MDC Gama Caldeira (Funchal; Hospital Dr. Nélio Mendonça); B Gama, CS Rodrigues, A Cabral, A Silva (Horta; Hospital da Horta, E.P.E.); E Borges, J Cassiano Neves, R Bernardino, P David Santos, J Secchi (Lisbon; Centro Hospitalar Lisboa Norte); M Nunes, D Tavares, M Cruz , C Quintela, C Cardoso, IM Lourenço (Matosinhos; Unidade Local de Saude de Matosinhos - Hospital Pedro Hispano); D Vaz Acosta, P Rego Ponte, R Santos Pereira (Ponta Delgada; Hospital do Divino Espírito Santo); H Capote, MB Mourato, T Mogne, N Andrade, G Fialho, F Valente Costa Pinto, B Cordeiro, M Brito, G Santos, D Rosado, C Costa, N Pratas (Portalegre; Hospital Doutor José Maria Grande); J Dias-Ferreira, AL Carreira-Marques, R Ribeiro Dias, B Carvalho, M Gomes, C Soares-Aquino, F Gomes, S Barbosa Castelo Branco, C Coutinho, JP Vieira de Sousa, D Atouguia (Porto; Centro Hospitalar e Universitário de São João); L Cidade Costa (Porto; Hospital da Prelada); D Silva, P Correia, C Henriques, AM Pinheiro Pereira, J Marques Antunes (Santa Maria da Feira; Centro Hospitalar Entre o Douro e Vouga); H Devesa, R Barradas, S Fortuna Martins, N Marcos, A Jarimba, B Louro, L Rodrigues Madeira, AR Lourenço (Santarem; Hospital de Santarem); A Ferreira, A Abreu da Silva, D Stoian , M Ferreira (Santiago do Cacém; Hospital do Litoral Alentejano); R Branquinho, JC Domingues, MI Seixo, R Lalanda, C Bôto (Tomar; Centro Hospitalar Médio Tejo); J Fernandes, P Laranjo , M Reis, I Borges da Costa, C Assis, B Lopes Patrício, NM Freitas Oliveira (Vila Franca de Xira; Hospital Vila Franca de Xira); M Carvalho, J Mendes, C Macedo Cardoso de Oliveira, B Freire, R Pinheiro Duque (Vila Nova de Famalicao; Centro Hospitalar do Medio Ave); B Vieira, U Fernandes, A Dupont, J Ribeiro, R Vaz Pereira (Vila Real; Centro Hospitalar de Trás-os-Montes e Alto Douro, E.P.E.).

Romania: É Gáspár (Brasov; Regina Maria); A Chitul, C Bezede, E Ciofic, D Cristian (Bucharest; Coltea Clinical Hospital); EA Toma, IM Matache, O Enciu, B Bogdan-Gabriel (Bucharest; Elias Emergency Hospital); I Negoi, C Ciubotaru, I Tanase, C Dina, VM Negoita, A Perja (Bucharest; Emergency Clinical Hospital Bucharest); R Drasovean, A Trif, D Misca, C Hossu, I Imihteev (Cluj-Napoca; Medicover Hospital Cluj).

Russian Federation: V Kakotkin, M Agapov, V Budyakova, S Dos Santos Rocha Ferreira, R Senin (Kaliningrad; Immanuel Kant Baltic Federal University, Regional Clinical Hospital); A Bedzhanyan, A Sumbaev, K Petrenko, E Bedzhanyan, E Tyurina, R Azimov, P Glushkov, K Shemyatovsky , S Husanov, A Sidorova (Moscow; Petrovsky National Research Centre of Surgery); G Yarovenko, E Shestakov, O Lisin, A Arustamyan, S Katorkin (Samara; Hospital Surgery Clinic of Samara State Medical University); J Sidorovskaia, K Cholah, I Cholah, D Kurochka (Simferopol; Municipal Emegency Hospital No.6); V Ten, Y Kudryavcev (Yuzhno-Sakhalinsk; Private healthcare institution ‘RZD-Medicine’).

Serbia: J Juloski, V Cuk, V Cijan, L Milic (Belgrade; Zvezdara University Medical Center).

Slovenia: JA Košir, J Grosek, A Tomazic, T Košir Božič (Ljubljana; University Medical Centre).

Spain: H Aguado López, F Ruescas, A García Marín, M Scortechini, M Jurado Román , A Sanchez Gallego (Albacete; Hellín Hospital); E González Marín, S De la Cruz Ahufinger, M Mateu, MJ Medina, MDM Martí-Ejarque, R Soliva Domínguez, L Ruiz-Villa, E Montalbán Martínez (BARCELONA; Hospital Universitari Sagrat Cor); A Torroella, C Ginesta, G Cárdenas Rivera, VE Gonzabay, JD Acevedo Parrales (Barcelona; HM Nou Delfos); M Canals Sin, A Lombardero, B Capdevila Vilaró, M Carbonell Pradas, ME Muñoz Fernández , RA Hernandez Rodriguez, I De Haro Jorge, M Riba Martínez, L Tapia Moral, M Coronas Soucheiron, P Palazon Bellver, L Ortega Lechuga, X Tarrado, A Domenech Plana, J Prat-Ortells, M Bejarano Serrano, M Cuesta Argos, MP Martin Gimenez, SG Laura, R Ripoll i Palmés (Barcelona; Hospital Sant Joan de Deu); A Sainz Lete, JC Zevallos-Quiroz, D Gómez, B Estraviz, JM De Francisco Rios, J Barrutia Leonardo, M González de Miguel (Bizkaia; Hospital Urduliz); RL Ferlini, M Ortega Escudero, Y Galvañ Félix, C Hernandez Diaz, J Montero García (Burgos; Hospital Universitario de Burgos); Á Fernández Camuñas, EP Garcia Santos, FJ Redondo Calvo (Ciudad Real; Hospital General Universitario de Ciudad Real); M Estaire Gómez, RJ Castro Lara, A Ramos Bonilla, L Rodríguez Gómez, M Marqueta De Salas, A Alvarez Cuiñas, FM Bujalance Cabrera, MD Cancelas, A García Domínguez, G Chamoso Mialdea, EP Cagigal Ortega, D Enjuto, I Cervera (Leganés; Severo Ochoa University Hospital); R Villalobos Mori, Y Maestre González, C Gas, L Codina Corrons, C Semeraro (Lleida; Hospital Universitari Arnau de Vilanova); J García-Quijada, TW Jorgensen, L Marquez (Madrid; Hospital Central de la Cruz Roja San Jose y Santa Adela); MJ Peña Soria, N Tabatabaian , JL Garcia galocha, D Fra Corral, L Sante Serna (Madrid; Hospital Clinico San Carlos); M Diez Alonso, C Vera Mansilla, L Casalduero, S Soto Schütte, Y Allaoua (Madrid; Hospital Universitario Principe de Asturias); E Gutierrez, C Zapata Syro, F Prieto La Noire, MDM Olmedo Reinoso, S Salido, N Chavarrias, M Vicario Bravo, L Asensio Gomez, R Abad, A Gegúndez Simón, PC Arteaga Asensio (Madrid; Hospital Universitario la Paz); AM Minaya Bravo, E González , A Galvan, C Guijarro Moreno, G De la Peña González, A Sánchez Gollarte, A Robin Valle de Lersundi, MÁ García Ureña (Madrid; Hospital del Henares); JL Rodicio Miravalles, AA Suárez Álvarez, DW Silva-Cano, G Martínez Izquierdo, P Del Val Ruiz, M Moreno Gijon, S Amoza Pais, GP Ibero Casadiego , E López-Negrete Cueto, J Carrizo, S Sanz, R Rodríguez-Uria, A Cembellin, G García-Santos, A Fraile (Oviedo; Hospital Universitario Central de Asturias (HUCA)); D Córdova García, L Jiménez, J Martin Fernandez, R Alvarado Hurtado, AM Minaya Bravo, R Díaz Pedrero, N Cobeño Tamayo, V Ongil Rodríguez (Rivas-Vaciamadrid, Madrid; Hospital Universitario HM Rivas); F Aguilar del Castillo, Á De Jesús Gil, S Borrego Canovaca (Sevilla; Hospital Universitario Virgen del Rocio); JR Naranjo Fernández, Z Valera Sanchez, R Perez, M Infantes Ormad, M Sánchez Ramirez (Seville; Hospital Universitario Virgen Macarena); C Leal Ferrandis, C Esteo Verdu, S García López, J Febré, B Cuneo (Valencia; Hospital Arnau de Vilanova); C León-Espinoza, E Martí Cuñat, G Pou (Valencia; Hospital Clínico Universitario de Valencia); C Jezieniecki, S Alonso Marcos, A Vazquez Fernandez, J Beltrán de Heredia, B De Andrés-Asenjo, D Baños Méndez, JC Garcia Vera, M Ruiz Soriano, E Redondo, R Martínez Díaz , T Gómez Sanz, P Artigot, C Infante, C Ferreras García, LR Cabezudo, G Cabezudo, M Lainez Escribano, M Rodriguez-Lopez, H Nuñez Del Barrio, A Romero de Diego (Valladolid; Hospital Clínico Universitario de Valladolid); A Vazquez Melero, M Camuera, I Herrero, D Garcia López de Goicoechea, M Sánchez-Rubio (Vitoria-Gasteiz; Hospital Universitario Araba); MDP Cebollero, JL Blas Laina (Zaragoza; Hospital Royo Villanova); V Duque Mallén, N Sánchez Fuentes, P Sancho Pardo , I Gascon Ferrer, MÁ Dobón Rascón, T Gimenez Maurel, J Chóliz, S Saudí-Moro, S Paterna -Lopez, A Martinez German, D Aparicio-López, MÁ Gascón Domínguez, P Royo Dachary (Zaragoza; Hospital Universitario Miguel Servet).

Sweden: M Zaigham, A Al Mukhtar (Malmö; Skåne University Hospital); M Nikberg (Vasteras; Västmanlands Hospital Västerås).

Switzerland: D Fenner, D Salinovic (Frauenfeld; Spital Thurgau AG); X Papazarkadas, TV Pham, C Brasset, A Litchinko, F Ris, C Golliez, M Chevallay (Geneva; Geneva University Hospitals); J Gass, J Mühlhäusser, J Metzger, A Scheiwiller (Luzern; Luzerner Kantonsspital); A Tampakis, C Riboni, U Dietz, E Brolese, C Seiler, M Kalisvaart, JN Marx, L Eisner (Olten; Kantonsspital Olten); G Peros, F Solimene, M Gramellini, A Lareida, M Adamina, E Betz, L Dubs, K Geiger-Timm, L Gantner, N Seeger, K Richetti, K Hofmann (Winterthur; Kantonsspital Winterthur); MA Schneider, D Gero, K Lehmann, P Limani, S Hügli, S Gerdes, F Mazzola, A Hiller (Zurich; University Hospital of Zurich).

United Kingdom: ZA Fozo, A Shoker, KR Rahman, MW Saqib, R Faisal, CS Ong, A Pillai, H Unwin, A Huws, M Maybury, H Ejaz, E Daketsey, AK Lala (Bangor, North Wales; Ysbyty Gwynedd); MAK Sarker, B Chkir, MR Peris, S Khan , MA Tahir, N Sharma, R Doherty, H Alhusaini, MF Butt, H Afzal, S Handa, N Maharjan , A Mostafa, G Lee, CK Lim, A Anand, A Krishna, WT Yew, Y Lu, R Hall (Barrow in Furness; Furness General Hospital); E Mohammed (Basildon; Basildon University Hospital); F Georgiades, S Karim, K Rajaratnam (Bedford; Bedford Hospital); J Abbasy, A Bibi, S Karandikar, L Johnstone , N Fazili (Birmingham; Heartlands Hospital); A Singh, A Athanasiou, H Lidbetter, J Siby, M Kaur, A Fatima (Brighton; Royal Sussex County Hospital); SM Reddy, A Campbell, A Cardoso Almeida, K Smith, CJ Bradshaw, K Tambudze, H Delacave, I Norman (Bristol; Bristol Royal Hospital for Children); M San, S Babu, S Midya, H Bradly, S Tontus, H Chauhan, R Jurdon, M Corcos , E Jose (Camberley; Frimley Health NHS FT - Frimley Park); N Eardley, B Davies, M Ransome, S Ahmed (Chester; Countess of Chester Hospital); S Suresh, A Bavaharan, R Batir, R Sato (Colchester; Colchester Hospital University); N Chidumije, M Ahmed, TY Kwan, F Olaniru, I Parwaiz, LP Cheng (Coventry; University Hospitals Coventry and Warwickshire NHS Trust); N Gokhare Viswanath, K Nanayakkara, A Tibude (Derby; Royal Derby Hospital); O Olajumoke, M Shams, D George, A Amin, M Kausar, S Sellahewa (Dudley; Russell’s Hall Hospital); H Kamal, A Kamal, M Kamal (Dundee; Ninewells Hospital); O Pryer, H Sagar, B Lulham-Robinson, S Dawo, A Nada (Durham; University Hospital North Durham); R Bethune, G Chillarge, AM Myintmo, M Horga, L Andreski, E Ruiz-Daum , T Finlay, I Rakshit (Exeter; Royal Devon and Exeter Hospital); J Bryan, D Joshi, R Marlin, M Battili , YL Aung, L Zeng, S Mathew, I Njere, S Gopaul, A Abbas (Great Yarmouth; James Paget Univeristy NHS Foundation Trust Hospital); G Singh, M Asarbakhsh, S Staight (Huddersfield; Huddersfield Royal Infirmary); R Govindaraju, M Quaunine, S Yassin, A Wilkins, J Walshaw, L Chang, A Mahendran, F Hammett, D Fairbrass, UA Kalu, E Dexter, T Nadeem, N Karunaratne, T Lo, M Pellen (Hull; Hull University Teaching Hospitals NHS Trust); SH Sarwary, J Otote, D Anbu, H Islam, N Morricone (Ilford; King George Hospital); K Lee, E Gimson, M Wilson, C Chiam (Larbert; Forth Valley Royal Hospital); M Solkar, M Bautista, NS Blencowe, A Gupta, J Sutcliffe, A Ahmad, A Peckham-Cooper (Leeds; Leeds Teaching Hospitals); E O’Connell, KE Dey, R Lunevicius, MM Barakat, GR Goodwin, B Devkaran, IC Nzenwa, A Pilavas (Liverpool; Aintree University Hospital); K Bananis, A Alamin, S Bennett , MD Barcelona, A Sharp, F Soggiu (London; Ealing Hospital); E Baili, H Ebied, A Botha, M Haghighat Ghahfarokhi, MMT Youssef (London; Guy’s and St Thomas’ Hospitals); K Theodoropoulou, A Quddus, R Hegy, M Mahran, A Ghanbari (London; Homerton University Hospital); P Kapsampelis, T Chouari, J Saunders, C Boven, I Gerogiannis (London; Kingston); N Karthikeyan, C Karagianni , E Spanoudakis (London; Queen Elizabeth Hospital, Woolwich); H Younus, R Ben Hmida, D Eaton (London; The Whittington Hospital); C Seet, R Bradley, R Roberts (London; University Hospital Lewisham); FN Amir, M Durrani, S Khan, A Tahir (Macclesfield; Macclesfield District General Hospital); A Khalil, E O’Neill, S Ingley, V Bill, P Wilson, M Elmousili, E Chin , C Alphonse, P Suresh, L Devi, C Shelton (Manchester; Wythenshawe Hospital); S Bugren, M Abdelreheem, MMR Azzuz, F Gareb (Margate; Queen Elizabeth the Queen Mother Hospital Margate); DJ Dhillon, MF Khan, B Peter, I Fagiri , T Harris , P Thambi (Middlesbrough; James Cook University Hospital); M Tomlinson, C Hidalgo Salinas, A Mwanjoka , M Catterall, B Ali (Morecambe; Royal Lancaster Infirmary); S Tingle, K Waddell, F Peters, T Akharaekpanya, S Robinson (North Shields; Northumbria NHS Hospital Trust); NA Binti Yusri, A Ashiru, SA Chowdhury , J Reilly, S Malek, S Kumaran, A Doghaim, ABA Al-Hajjaj, LR Chieng, ZY Wong, R Olatunji (Nottingham; Queens Medical Centre); J Bundred, B Down, A Ang, KA Shamiyah, G Bond-Smith (Oxford; John Radcliffe Hospital); M Elmesalmi (Portsmouth; Queen Alexandra Hospital); S Monkhouse , O Mohamed, B Robertson-Jones, P Patel, BZ Hao (Redhill; East Surrey Hospital); S Ghattas, N Beharry, A Maraqa, N Uttam, S Rafiq, A Abdelhamid, E Mazumdar, M Dyer, MEEA Abdelsalam, B Johnson, M Abdelkarim, A Murtada, MMS Tora, Z Azhar (Rhyl; Glan Clwyd Hospital); O Whitehurst, K Bhatti, S Silvestre, LN Bin Aizan, A Patel (Salford; Salford Royal Hospital); Z Khan, A Maqsood-Shah, O Webster, H Reilly, S Boyes (Sheffield; Sheffield Teaching Hospital NHS Foundation Trust); S Bandyopadhyay, B McDermott, H Kynaston, B Neall, M West, G Hart, R Titcombe , H Kaur, A Ekerin, A Demetriou, N Harrison, JJQ Chen (Southampton; Southampton General Hospital); M Hammoda, L Robine-Durnell, E Mansour, A Evans, E Baker (Swansea; Morriston Hospital Swansea); I Abdullah, O Osunlusi (Walsall; Walsall Manor Hospital).
